# CO-tolerant hydrogen oxidation on Pt enabled by ultrathin silica overlayers

**DOI:** 10.1039/d6ey00101g

**Published:** 2026-08-24

**Authors:** Christos Englezos, Marco Altomare, Guido Mul, Bastian Mei, Georgios Katsoukis

**Affiliations:** a MESA+ Institute of Nanotechnology, Department of Chemical Engineering, Faculty of Science and Technology, University of Twente Drienerlolaan 5 7522 NB Enschede The Netherlands g.katsoukis@utwente.nl; b Fakultät für Chemie & Biochemie, Technische Chemie - Energie und Umweltrelevante Katalyse, Ruhr-Universität Bochum Germany bastian.mei@rub.de

## Abstract

Low-temperature electrochemical hydrogen compression can combine H_2_ compression and purification, but CO impurities in practical H_2_ feeds rapidly poison Pt anodes. Here, we use sputter- and electrodeposited Pt on Ti disk model electrodes to assess whether ultrathin polydimethylsiloxane (PDMS)-derived amorphous silica overlayers can improve CO tolerance during the hydrogen oxidation reaction (HOR). Fourier-transform infrared reflection–absorption and low-energy ion-scattering spectroscopy confirmed conformal and amorphous silica overlayer formation on Pt. Nominal thickness-dependent measurements revealed a narrow activity-protection window within the sub-10 nm regime. 2–3 nm silica overlayers provided limited CO tolerance, whereas increasing the nominal thickness from 7 to 8 nm strongly suppressed HOR activity. Benchmarking against active electrochemical regeneration showed that bare Pt operated at 0.15 V *vs*. RHE with 15 s CO-stripping pulses at 0.85 V *vs*. RHE delivered a 1.8 times higher time-averaged HOR power density than silica-coated Pt. However, the pulses introduced an 8% energy-efficiency loss and rely on repeated high-potential cycling that accelerates electrode degradation. Importantly, under fixed-current operation, silica-coated Pt reduced the cumulative HOR energy demand under CO contamination by a factor of up to 3.6. These results establish ultrathin silica overlayers as a promising interfacial design strategy for CO-tolerant hydrogen oxidation and motivate translation to gas-diffusion electrodes (GDEs), where homogeneous and selective coating of porous electrodes remains the key challenge.

## Abstract 2

Ultrathin amorphous SiO_*x*_ overlayers improve Pt tolerance to CO poisoning during the hydrogen oxidation reaction compared with uncoated Pt.

## Graphical abstract

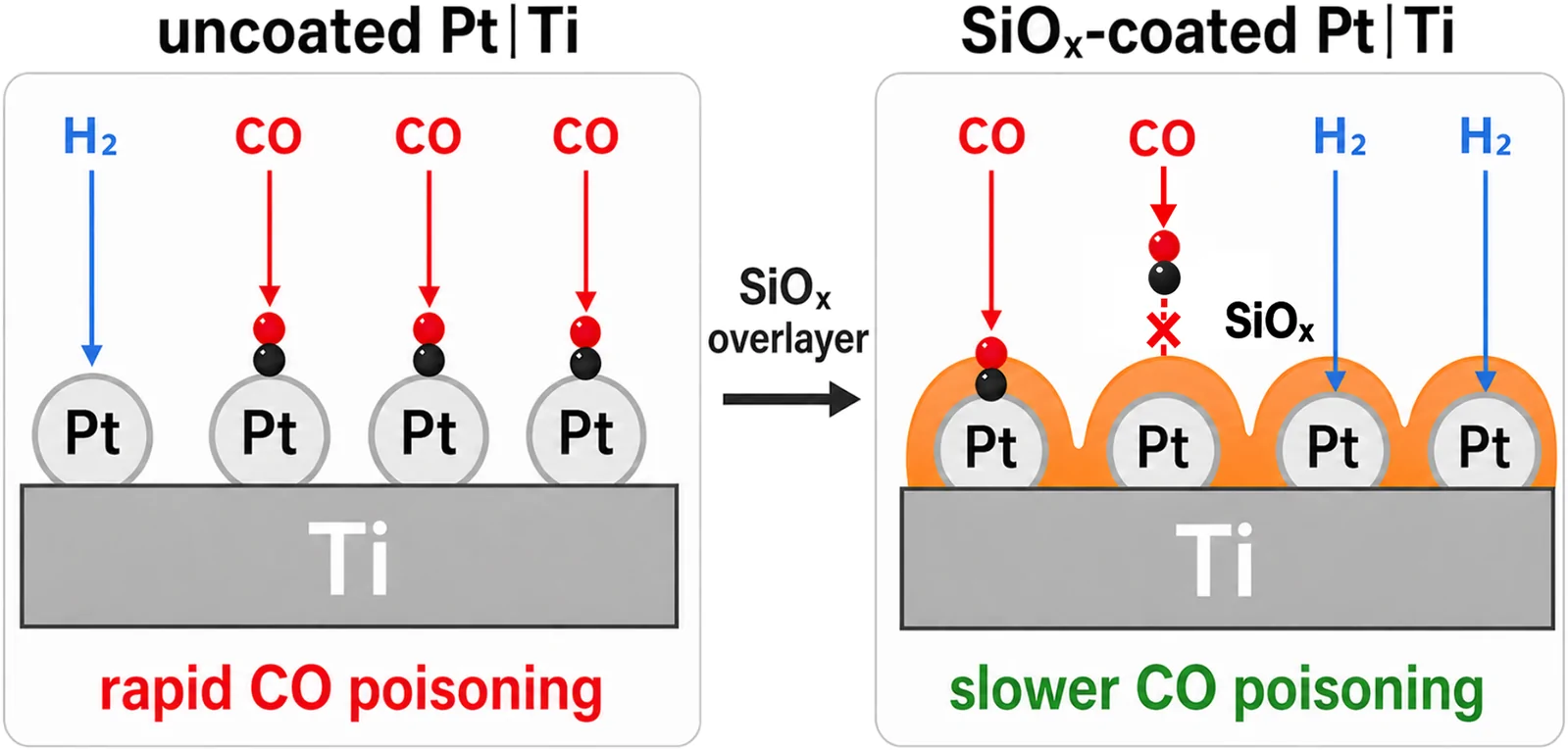

**Broader context**

Today, H_2_ is still largely produced from natural gas, coal, reformate, and industrial by-product streams. Long-term net-zero pathways will still retain fossil-derived H_2_ with carbon capture alongside growing electrolysis. H_2_ compression and purification will therefore remain coupled challenges, especially for only partially purified, recovered, or recycled H_2_ streams. Electrochemical hydrogen compression is attractive because it can pressurize H_2_ while selectively separating it from impure feeds, but this requires anodes that remain active in the presence of impurities. Pt remains the benchmark H_2_ oxidation catalyst because of its high activity at low overpotential. Carbon monoxide is especially relevant for carbon-derived H_2_ and purification-breakthrough events because it strongly binds to Pt and blocks the sites required for H_2_ oxidation. Catalyst replacement is difficult because alternative materials and alloys or engineered catalyst facets often improve CO tolerance at the cost of activity or stability. A practical materials direction is therefore to retain Pt and control molecular access to its surface. This work positions ultrathin catalyst coatings as a route to impurity-tolerant H_2_ oxidation electrodes, with conformal coating of porous electrodes emerging as the key scale-up challenge.

## 1. Introduction

Rising energy demand and climate targets are accelerating the transition from fossil fuels to low-carbon energy systems.^1^ Hydrogen (H_2_) is central to this shift as both a major industrial feedstock (*e.g.*, refining, NH_3_ and methanol synthesis) and a prospective energy carrier. However, given the low volumetric energy density of H_2_, most storage, transport, and fueling concepts require compression.^2,3^ Conventionally, this pressurization is provided by mechanical compressors. Electrochemical hydrogen compression (EHC) has therefore emerged as an attractive alternative, particularly for electrolyzer-integrated and renewable electricity-driven processes, because it can, in principle, combine compression and purification in a compact device without moving parts.^3–7^ However, these advantages in practice require durable operation at high current densities, large pressure differentials, and sufficient water management.^8–11^

In practice, PEM-based EHC is classified as low-temperature (LT) EHC and high-temperature (HT) EHC. LT-EHC typically employs perfluorosulfonic-acid membranes, such as Nafion, prone to membrane hydration and hydrogen back-diffusion, while also being more susceptible than HT-EHC (based on phosphoric-acid-doped polybenzimidazole membranes) to performance losses in CO-containing feeds.^5,8,12^ Nevertheless, single-stage LT-EHC compression up to 1000 bar has been reported.^7,13^ The competitiveness of LT-EHC technology is governed by two coupled requirements: high energy efficiency (low polarization losses) and high throughput per cell (high current density). However, H_2_ feeds can contain trace contaminants, such as CO (tens to tens of thousands of ppm), hydrocarbons, and H_2_S (tens of ppm) from upstream reforming or incomplete pre-purification, that can poison the commonly employed Pt anodes even at ppm level concentrations.^10,14–16^

Several strategies have been explored to improve the CO tolerance of Pt-based hydrogen oxidation reaction (HOR) catalysts. Periodic CO stripping at anode potentials above +0.8 V *vs.* SHE can restore activity, but introduces intermittent efficiency penalties and raises durability concerns due to repeated positive-potential cycling (Pt oxidation/reduction, dissolution, and particle growth *via* redeposition). These constraints motivate the use of CO-tolerant anodes that enable continuous operation.^5,14,17^ Alloying Pt with secondary metals such as Pd, Ru, Sn, or Mo was shown to weaken CO adsorption and promote its oxidative removal.^18–24^ Pt–Ru alloys, for example, operate *via* a bifunctional mechanism in which Ru supplies oxygenated species that facilitate CO oxidation at lower potentials. Pt–Sn and Pt–Mo alloys show analogous effects under reformate hydrogen conditions. This alloying is also an established strategy for enhancing HOR/hydrogen evolution reaction (HER) activity and CO tolerance by tuning hydrogen- and hydroxyl-binding energies through electronic, strain, and bifunctional effects.^25,26^ However, the resulting activity and durability depend strongly on the alloy composition and operating conditions, as less noble alloying elements may undergo dissolution or surface segregation.^27^ Crystal-facet engineering offers another route, as CO adsorption energies differ among Pt facets.^28–30^ Close-packed Pt(111) binds with CO more weakly than more open surfaces such as Pt(100) or Pt(110), while retaining high HOR/HER activity. In practice, though, surface reconstruction during electrochemical operation tends to erode these advantages, and surface-defined Pt electrodes usually converge to similar poisoning behavior over extended operation as polycrystalline Pt electrodes.

An increasingly attractive strategy for mitigating catalyst poisoning is the use of ultrathin, semipermeable electrocatalyst overlayers, also called membrane-coated electrocatalysts (MCECs).^31–33^ Various inorganic overlayers such as Cr_*x*_O_*y*_H_*z*_,^34^ CrO_*x*_,^35^ VO_*x*_,^36^ TiO_*x*_,^37^ SnO_2_^38^ and SiO_*x*_,^39–43^ have been proposed in the literature to tune interfacial transport and selectivity in HOR/HER reactions, with SiO_*x*_ being most frequently employed. SiO_*x*_-coated Pt MCECs preserve the HER at buried Pt–SiO_*x*_ interfaces through selective proton transport with exclusion of larger species like Cu^2+^.^44^ These MCECs promote CO and methanol oxidation *via* silanol-assisted bifunctional effects and lower CO oxidation onset potentials,^45^ as well as suppress the chlorine evolution reaction (CER) while maintaining the oxygen evolution reaction (OER) by limiting chloride permeation.^46^ Although these studies establish the conceptual proof of SiO_*x*_ overlayers for interfacial selectivity, they do not show whether such coatings remain effective for LT-EHC Pt anodes when exposed to practically relevant CO-containing hydrogen streams. Sustained HOR throughput and preserved HOR catalytic activity of buried (*i.e.*, protected) Pt compared to bare Pt are the key performance metrics. In particular, the coupled effects of overlayer thickness and Pt substrate morphology on (i) the HOR activity–CO tolerance trade-off, (ii) the associated H_2_ transport resistance through the silica barrier, (iii) the stability of the protective effect under prolonged poisoning, and (iv) the resulting energetic penalty during CO contaminated operation have not yet been examined.

In this work, we address these outstanding questions by preparing ultrathin polydimethylsiloxane (PDMS) derived SiO_*x*_ overlayers on two Ti-supported Pt electrodes, namely planar sputtered Pt and nanostructured electrodeposited Pt. We systematically evaluate how overlayer thickness and electrode architecture govern HOR activity, CO tolerance, coating stability, and energy demand. Using scanning electron microscopy (SEM), infrared reflection–absorption spectroscopy (IRRAS), and low-energy ion scattering (LEIS), we investigate the morphology, chemical conversion, and conformal coverage of the silica overlayers, while rotating-disk HOR measurements in pure H_2_ are combined with a resistances-in-series transport analysis to quantify the H_2_ permeability enabled by the SiO_*x*_ layer. CO poisoning is examined under both highly aggressive (17 000 ppm CO) and industrially relevant (1700 ppm CO) CO/H_2_ feeds using potentiostatic and chronopotentiometric protocols, with which the energy demands of both coated and uncoated Pt anodes were estimated, and compared to their HOR operation in a pure H_2_ atmosphere. Together, these results aim to provide a quantitative basis for designing CO impurity-tolerant Pt anodes for LT-EHCs.

## 2. Experimental

Titanium Grade 23, also known as Ti-6Al-4V ELI (extra low interstitials), is used as the Ti disk substrate. The disks were mechanically cut to have an outer diameter (OD) of 5 mm and a thickness of 4 mm. The Ti disks were hand-polished and etched each time prior to any further coatings. First, using SiC abrasive paper, the disks were hand-polished on one of the two circular sides with the following order and grit sizes: P400 for 1 min, P1000 for 1 min, and P2000 for 2 min. After rinsing with MilliQ water, the disks were immersed in 10 mL of freshly prepared aqua regia (hydrochloric acid ACS reagent, 37% and nitric acid ACS reagent, 70%) and left under a fume hood for 2 hours. Then, they were rinsed with MilliQ water and immersed in a beaker with 10 mL Milli-Q water. The beaker was placed in the ultrasonic cleaner for 5 min. The disks were removed from the beaker, rinsed with Milli-Q water and dried under a N_2_ stream.

### 2.1. Magnetron sputtering

A 50 nm Pt thin film was prepared on the polished Ti disk substrates using a magnetron sputter coater (ATC Polaris, AJA International Inc., USA), equipped with a Pt target (99.99% purity, AJA International Inc., USA), powered by an RF power supply (Power source 0313GTC, T&C Power, USA). More details (*e.g.* Pt mass loading per unit surface, nominal thickness *vs.* sputtering time, and physicochemical characterization) can be found in previous work.^47^

### 2.2. Electrodeposition

The Ti surface was electrochemically cleaned prior to the Pt electrodeposition experiment. The mounted Ti disk was first cycled 30 times in aqueous 0.1 M HClO_4_ (ACS reagent, 70%, Sigma-Aldrich) within the range of 0.03–1.30 V *vs.* RHE at 500 mV s^−1^ and then it was rinsed with Milli-Q water. The Pt electrodeposition experiments were performed using a Bio-Logic VSP potentiostat with EC-Lab software. A 3-electrode setup was used: a polished and clean Ti disk as the working electrode, Pt foil as the counter electrode (Metrohm AG) and HydroFlex hydrogen as the reference electrode (Gaskatel GmbH). The electrochemical cell used is a simple batch cell filled with 10 mL of the aqueous Pt precursor solution. The solution consists of 5 mM chloroplatinic acid, H_2_PtCl_6_ (hydrate, ≥99.9% trace metals basis, Sigma-Aldrich) in 0.5 M aqueous sulfuric acid (ACS reagent, 95.0–98.0%, Sigma-Aldrich). A 2-step Pt electrodeposition protocol was applied,^48^ which consists of chronoamperometry at −0.25 V *vs.* RHE for 5 s and without a resting period in between, switched to +0.25 V *vs.* RHE for 15 min. Upon completion, the disk was unmounted, rinsed with Milli-Q water and dried under a N_2_ stream.

### 2.3. Spin-coating and UV-ozone treatment

A Pt|Ti disk was inserted into the slot of a custom Teflon spin-coating holder, rinsed with Milli-Q water, and dried under a N_2_ stream. The holder with the disk was mounted on the spin-coater (Laurell WS-650MZ-23NPP, Laurell Technologies Corporation) and rotation was started. At 500 rpm s^−1^, a rotation from 0 to 4000 rpm takes 8 seconds and when this point was reached, 200 µL of 5, 10, or 20 mg mL^−1^ of polydimethylsiloxane, PDMS (silicone oil, 100 mPa s at 20 °C, Sigma-Aldrich) in toluene (Assink Chemie B.V.), was manually dropped slowly using an automatic pipette, on the center of the disk. The total time taken for spin coating from starting the rotation (0 rpm) was 2 minutes and 30 seconds. Upon completion, the disk was removed from the custom holder and placed on a ceramic plate. An atmospheric drying step at 90 °C for 1 h followed, with a 30 min ramp from 23 °C to 90 °C (Nabertherm L3/11/B410, Nabertherm GmbH). After the atmospheric drying step, the plate with the disk was removed from the furnace and placed inside the UV-ozone curing chamber for 2 h (UV/Ozone ProCleaner 220-Plus, BioForce Nanosciences). After the curing, the disk was rinsed with Milli-Q water and dried under a N_2_ stream.

### 2.4. Atomic layer deposition

SiO_2_ films on Pt sputtered wafers (for the silica thickness calibration) were deposited using a Veeco Savannah S100 thermal ALD system (Veeco Instruments Inc., USA) at 200 °C under vacuum. Pure N_2_ was used as the carrier gas. Bis(diethylamino)silane (BDEAS; PURATREM, Strem Chemicals) was used as the Si precursor and O_3_ as the oxidant. BDEAS was pre-heated to 50 °C prior to delivery, while ozone was generated *in situ* from pure O_2_ using a Veeco ozone generator. Before SiO_2_ growth, the substrates were exposed to 10 O_3_-only pretreatment pulses.

### 2.5. Scanning electron microscopy and areal topography

The surface morphology of the prepared electrodes was analyzed by scanning electron microscopy (SEM). The pristine edep-Pt|Ti electrodes were characterized using a ZEISS MERLIN high-resolution scanning electron microscope (HR-SEM, Germany) operated at an accelerating voltage of 1.4 kV, while the 50 nm-Pt|Ti electrodes were characterized using a field-emission scanning electron microscope (FE-SEM, JEOL JSM-7610F). 3D areal surface topography/profilometry of the pristine Ti substrates was measured using a Sensofar S neox confocal microscope (Sensofar Metrology, Spain) and evaluated according to ISO 25178 after plane form removal. The plane form removal is a post processing operation that removes the form-curve of the sample surface and provides accurate estimation of the surface roughness and skewness.

### 2.6. FT-IR reflection–absorption spectroscopy

A Bruker V80v was used, which is equipped with a liquid nitrogen cooled medium-band (12 000–600 cm^−1^) MCT detector and, an MIR polarizer (KRS-5) inserted into an automatic polarizer rotational unit. A variable angle reflection accessory (Bruker A513/Q) was used and set to 60°. The resolution was set to 2 cm^−1^, and the aperture was set to 1.0 mm. IRRA spectra were acquired using the 1.0 mm aperture at an incidence angle of 60°. This corresponds to an elliptical sampling area of approximately 1.8 mm × 3.6 mm, covering about 33% of the total sample area. 200 scans were collected for each p- and s-polarization. A freshly prepared electrodeposited Pt|Ti disk served as a baseline. The final spectra were manually baseline corrected using a spline curve.

### 2.7. Low-energy ion scattering

All LEIS measurements were performed in-vacuum using a Qtac 100 spectrometer from IONTOF, after 10 min of O radical cleaning of the loaded sample's surface before any measurement. The ion gun is perpendicular to the sample's surface. A double toroidal electrostatic analyzer detects ions that are backscattered at an angle of 145° with respect to the sample's surface normal. Note that only ions are detected by the instrument. During the LEIS measurement, a primary beam of He ions scans over a 1.0 mm^2^ area, with a primary beam energy of 3 keV. The beam current was in the range of 2–4 nA and the acquisition time was under 4 min with an ion dose of around 3 × 10^14^ ions per cm^2^. Measurements were repeated twice on the same spot to verify that no significant damage was created on the surface.

### 2.8. Electrochemical measurements

A Bio-Logic VSP potentiostat with EC-Lab software was used. Electrochemical measurements were obtained using a standard three-electrode glass rotating disk electrode (RDE) cell using a WaveVortex rotator (Pine Research Instrumentation, Inc.) and 25 mL of electrolyte. The working electrode was a catalyst-coated disk with a 0.196 cm^2^ geometric area, used together with a Pt foil counter electrode (Metrohm AG) and a HydroFlex hydrogen reference electrode (Gaskatel GmbH). The electrolyte consisted of 0.1 M HClO_4_ prepared using Milli-Q water (18.2 MΩ cm) and perchloric acid (ACS reagent, 70%, Sigma-Aldrich). From the start of the protocol until completion of the chronoamperometry step, the electrolyte was continuously purged with pure H_2_ gas at 600 mL min^−1^. The working electrode was first equilibrated at open-circuit potential for 5 min under rotation at 400 rpm, after which the uncompensated resistance (*R*_u_) was determined using a current-interrupt method. Following a short stabilization period, five linear sweep voltammograms were recorded at a scan rate of 10 mV s^−1^ at increasing rotation speeds of 400, 900, 1600, 2500, and 3600 rpm. The electrode was then returned to open-circuit conditions at 2500 rpm and held for an additional 30 s before initiating chronoamperometry. During chronoamperometry, pure CO gas was introduced after 3 min to induce controlled CO poisoning at a flow rate of 10 mL min^−1^ for the 17 000 ppm CO tests, and 1 mL min^−1^ for the 1700 ppm CO tests. At the end of the poisoning step, both H_2_ and CO flows were stopped and rotation was turned off. CO stripping voltammetry was subsequently performed by cycling the electrode five times at 100 mV s^−1^ to remove the adsorbed CO species. For the pulsed CO poisoning-stripping the protocol is the same as before with only the CA being modified to introduce pulses and no CV stripping cycles were performed. The ECSA estimation was done using the hydrogen under-potential deposition method on Pt (*H*_upd_). ECSA cyclic voltammograms, performed only for the uncoated Pt samples in the range 0.03–1.3 V *vs.* RHE, were recorded at 100 mV s^−1^ for 20 cycles after a 15 min Ar purge at 130 mL min^−1^ of the 0.1 M HClO_4_ electrolyte. All experiments were carried out at room temperature, and all reagents were used as received. For each electrochemical experiment and replicate, a fresh Pt|Ti electrode was prepared. When a SiO_*x*_ coating was required, it was applied to the freshly prepared Pt electrode prior to any electrochemical testing or potential cycling.

## 3. Results and discussion

### 3.1. Characterization of SiO_*x*_-coated Pt

We compared two Pt electrode architectures: sputter-deposited (2D) Pt and electrodeposited (3D) Pt on 5 mm Ti disks. The Ti disk substrates exhibit a valley-dominated surface roughness with an average areal roughness^49^ of 1.07 m on the macroscopic scale and a finer 68 ± 7 nm at the microscale. This indicates a consistent valley-rich morphology across length scales (Fig. S1), a result of the manual-polishing of the Ti disks. The *H*_upd_-derived ECSA of the sputter-deposited Pt electrode (50 nm-Pt|Ti) is 0.4 cm^2^ and that of the electrodeposited Pt electrode (edep-Pt|Ti) is 2.8 cm^2^ (Fig. S2). The Ti disk geometric area is 0.196 cm^2^, thus the roughness factors are 2 and 14 for the 50 nm-Pt|Ti and the edep-Pt|Ti electrodes, respectively. The *H*_upd_-derived ECSA decreased from 2.8 cm^2^ for uncoated edep-Pt|Ti to 1.9 cm^2^ (Fig. S3) after deposition of the 10 mg mL^−1^ PDMS-derived SiO_*x*_ overlayer, corresponding to a reduction of approximately 32%.

**Fig. 1:**
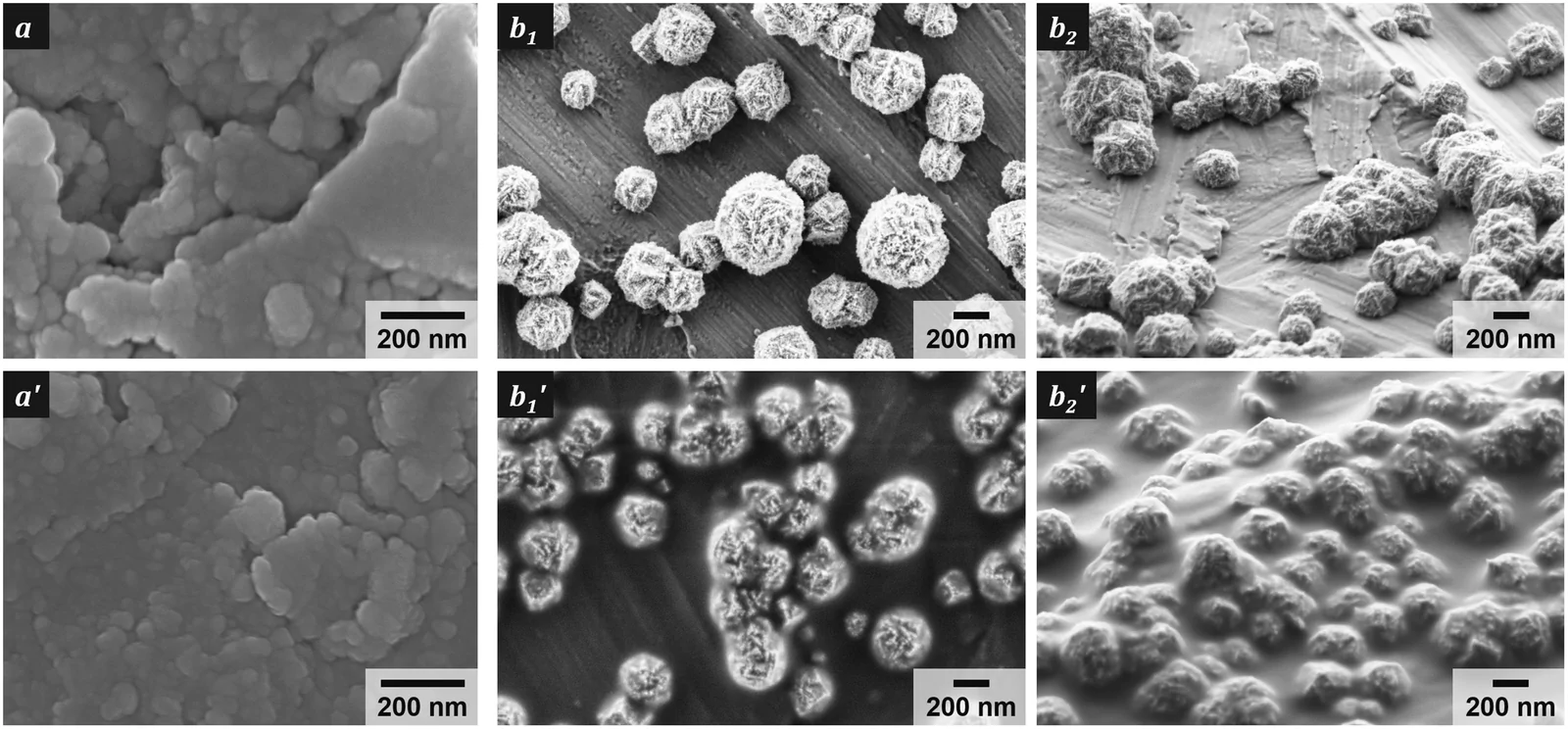
SEM images comparing uncoated and intermediate thickness (10 mg mL^−1^ PDMS) SiO_*x*_-coated Pt electrodes on polished Ti. Panel (a) shows the uncoated 50 nm-Pt|Ti electrode, while panel (a′) shows the corresponding SiO_*x*_-coated sample. Panels (b_1_) and (b_2_) show the uncoated edep-Pt|Ti electrode in plan view and tilted view, respectively, whereas panels (b_1_′) and (b_2_′) show the corresponding SiO_*x*_-coated edep-Pt|Ti electrode in the same views.

Fig. 1a shows the SEM image of the uncoated 50 nm-Pt|Ti electrode. The surface appears as a compact and continuous two-dimensional surface, with a fine granular morphology composed of coalesced Pt grains. Fig. 1a′ shows the corresponding intermediate thickness SiO_*x*_-coated 50 nm-Pt|Ti electrode. After coating, the overall surface morphology remains largely unchanged and the coating itself is not easily distinguished directly in SEM. Fig. 1b1 and b2 show SEM images of the uncoated edep-Pt|Ti electrode in plan view and tilted view, respectively. In contrast to the sputtered film, the edep-Pt|Ti electrode consists of isolated to partially agglomerated Pt particles with a porous flattened hemispherical morphology and characteristic lateral dimensions of approximately 200–600 nm. These Pt particles form a three-dimensional, island-like layer, leaving regions of the underlying Ti substrate uncovered. Quantitatively, 60–70% of the Ti surface remains exposed after Pt electrodeposition.

Fig. 1b1′ and b2′ show the corresponding intermediate thickness SiO_*x*_-coated edep-Pt|Ti electrode in plan view and tilted view, respectively, prepared by spin-coating a toluene solution of polydimethylsiloxane (PDMS), followed by atmospheric drying and UV–ozone curing. After coating, the Pt particle coverage and spatial distribution are retained, while the particle contours appear softened and the inter-particle regions become smoother. This is consistent with the Pt features being embedded beneath a continuous SiO_*x*_ overlayer.

**Fig. 2:**
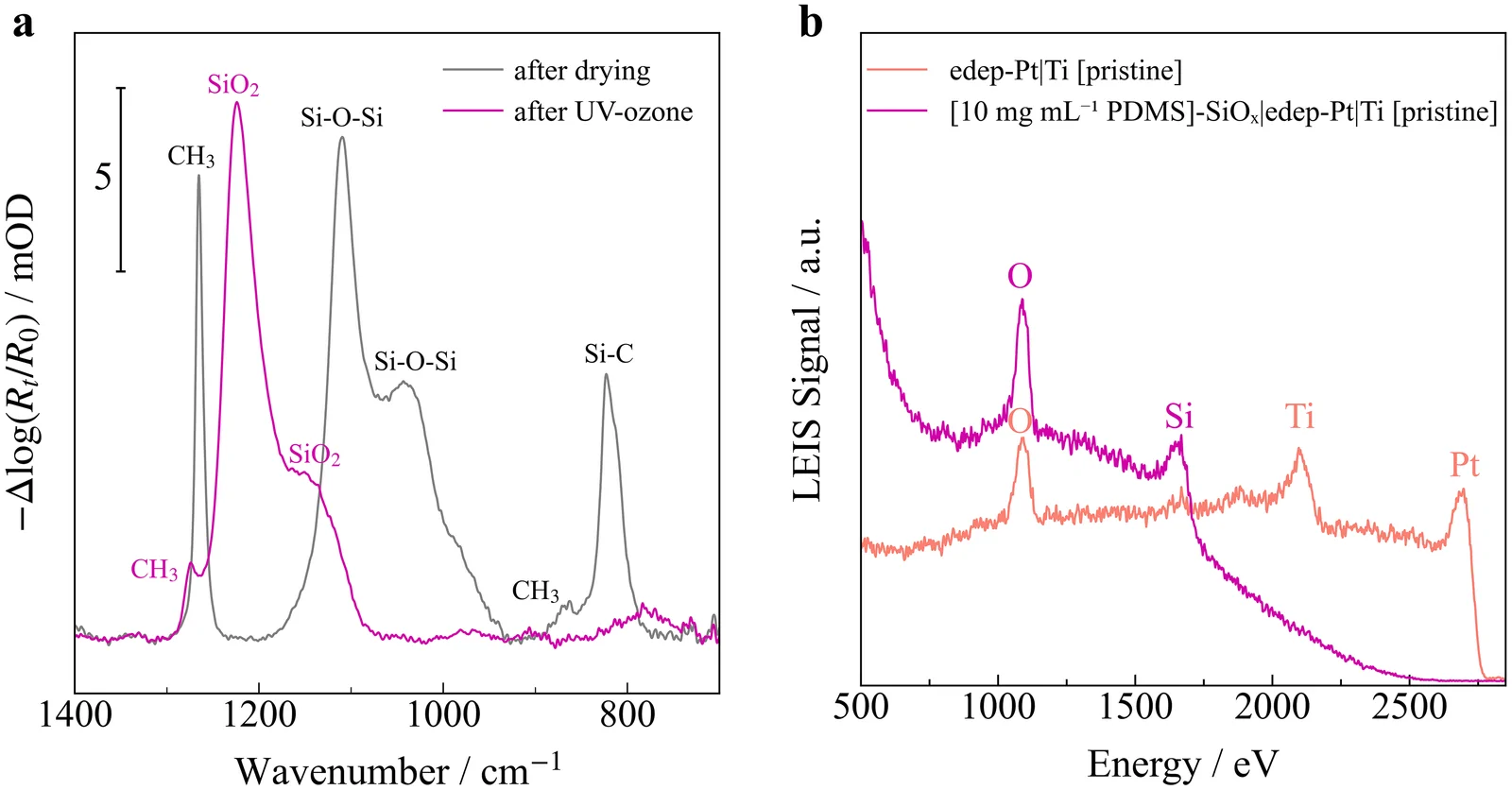
(a) Infrared reflection–absorption spectra (IRRAS) tracking the conversion of the spin-coated PDMS overlayer into an inorganic amorphous intermediate thickness SiO_*x*_ thin film (10 mg mL^−1^ PDMS) on edep-Pt|Ti. Spectra were collected using a 1.0 mm aperture at an incidence angle of 60°. mOD: milli-optical density units. (b) Low-energy ion scattering (LEIS) spectra for the pristine uncoated and intermediate thickness (10 mg mL^−1^ PDMS) SiO_*x*_ coated edep-Pt|Ti electrode. Measurements were performed using 3 keV He^+^ ions in ultra-high vacuum.

To assess the chemical composition of the PDMS-derived silica (intermediate thickness) on both the 50 nm- and edep-Pt|Ti electrodes, we used Fourier-transform infrared reflection–absorption spectroscopy (FT-IRRAS). As shown in Fig. 2a for the edep-Pt|Ti (representative), the spectrum recorded after spin-coating and drying is characteristic of PDMS, with the *δ*(CH_3_) deformation mode at 1260 cm^−1^, the Si–O–Si backbone bands at 1090 and 1020 cm^−1^, and the CH_3_ rocking and Si–C modes below 850 cm^−1^, in good agreement with reference PDMS spectra.^50–52^ Following UV–ozone curing, only a weak CH_3_-related residual signal remains at 1260 cm^−1^. The Si–O spectral region evolves into a broad, intense band centered at 1225 cm^−1^ (LO asymmetric vibration) with a lower-frequency shoulder near 1150 cm^−1^ (TO asymmetric vibration), characteristic of Si–O–Si stretching vibrations of bridging O species in amorphous SiO_2_/SiO_*x*_ films.^53,54^ The broad Si–O–Si bands are consistent with vitreous, predominantly amorphous silica, as IRRAS can distinguish ordered and vitreous silica structures.^55^

To evaluate the conformality of the SiO_*x*_ overlayer, we employed low-energy ion scattering (LEIS). As shown in Fig. 2b, the uncoated edep-Pt|Ti electrode exhibits the expected distinct peaks at 1.1, 2.2, and 2.7 keV, assigned to O, Ti, and Pt, respectively.^56–58^ The comparatively small O peak is attributed to native oxide and adsorbed oxygen species, likely enhanced by the O-radical cleaning step applied prior to LEIS analysis.^59^ After SiO_*x*_ deposition, the spectrum is dominated by a strongly increased O peak and an additional Si peak at 1.7 keV, while no Ti or Pt surface peaks are detectable above the noise level. The absence of Ti and Pt peaks indicates that these elements are buried beneath a conformal SiO_*x*_ overlayer.

### 3.2. Thickness dependent HOR and CO tolerance of SiO_*x*_-coated Pt

**Fig. 3:**
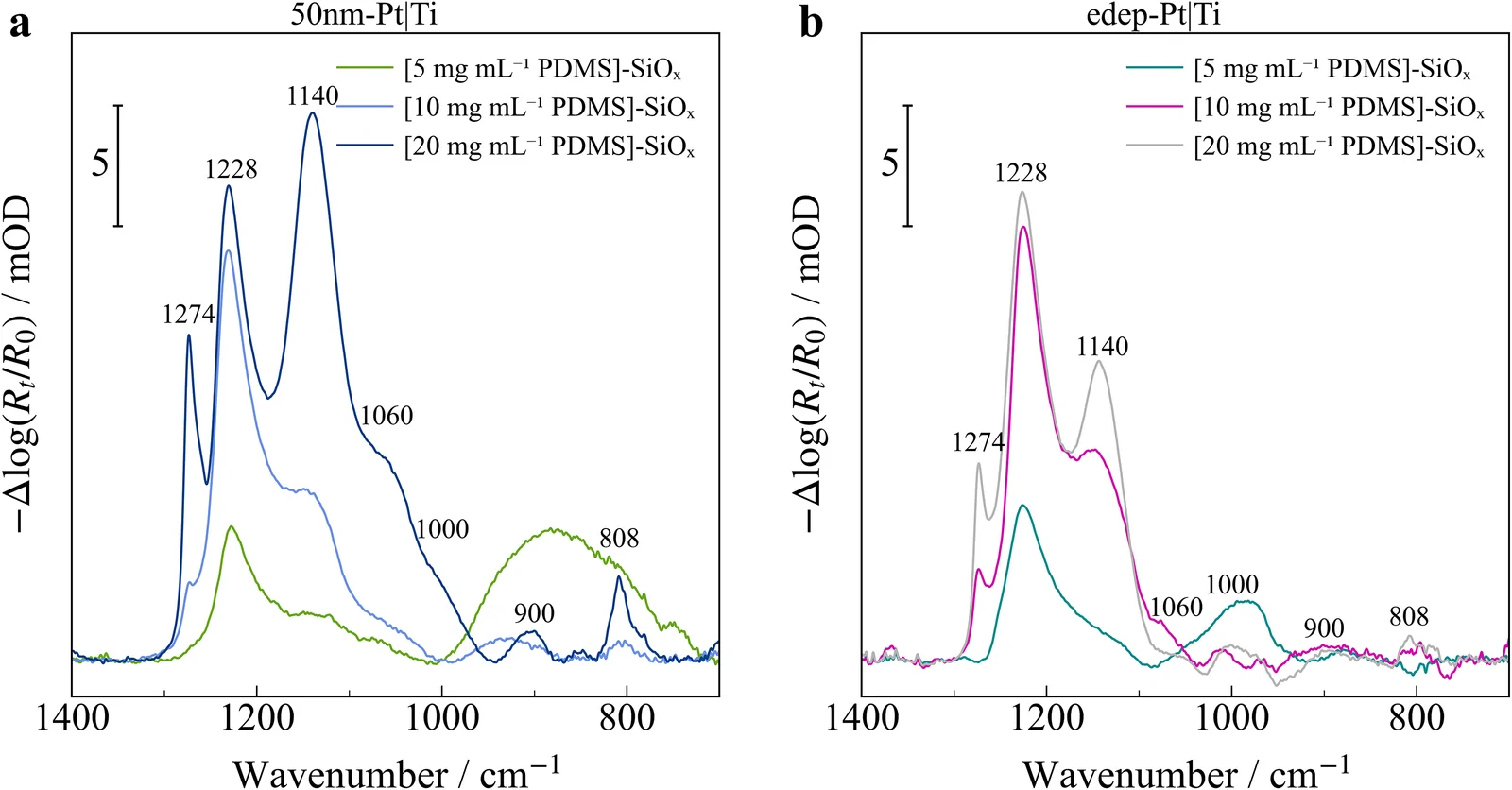
Infrared reflection–absorption spectra (IRRAS) of PDMS-derived SiO_*x*_ overlayers on 50 nm- and edep-Pt|Ti electrodes. Spectra were collected using a 1.0 mm aperture at an incidence angle of 60°. mOD: milli-optical density units. (a) 50 nm-Pt|Ti coated from PDMS precursor solutions of 5, 10, and 20 mg mL^−1^. (b) edep-Pt|Ti coated from PDMS precursor solutions of 5, 10, and 20 mg mL^−1^.

To vary the amount of PDMS deposited by spin-coating on the surface of both 50 nm-Pt|Ti and edep-Pt|Ti electrodes, we kept the dispense volume and spin speed constant, and varied only the PDMS precursor concentration in toluene (5, 10, and 20 mg mL^−1^). The corresponding FT-IRRA spectra are shown in Fig. 3. With increasing PDMS precursor concentration, the Si–O–Si stretching band at 1228 cm^−1^ increases as expected. In parallel, the residual methyl deformation band at 1274 cm^−1^ becomes more pronounced, indicating that UV-ozone treatment does not fully remove carbon-containing groups in thicker coatings. This suggests that oxidation becomes progressively limited in the deeper regions of the film.

A notable deviation is observed for the 20 mg mL^−1^ PDMS-derived coating: the 1228 cm^−1^ Si–O–Si band shows little further increase, whereas the 1140 cm^−1^ band becomes comparatively more intense. Such a shift in the relative longitudinal optical/transverse optical contributions has been associated with increased micro-structural disorder in sol–gel-derived silica networks.^60^ Here, this behavior coincides with a much stronger residual methyl band at 1274 cm^−1^, being consistent with incomplete conversion and a more heterogeneous coating at the highest loading. Since all PDMS coated samples were cured under UV for 2h, a higher curing duration applied for the 20 mg mL^−1^ PDMS-derived coating, could improve the conversion of PDMS to silica and reduce the amount of residual C.

The nominal silica-layer thickness (*l*_SiO_*x*__) was estimated for the samples prepared by varying only the PDMS concentration by calibrating the IRRAS LO asymmetric stretching band of SiO_2_ against a series of atomic layer deposited SiO_2_ reference films of known thickness, as confirmed by X-ray reflectivity (Fig. S4 and S5), and the resulting values are listed in Table 1. The applicability of this calibration to the edep-Pt|Ti substrates was independently verified using ALD-SiO_2_ (Fig. S6). These thicknesses should be regarded as nominal, comparative values only, since the actual SiO_*x*_ thickness on the Pt|Ti electrodes is expected to vary with substrate roughness, Pt morphology and surface area, which affect the fraction of dispensed volume retained upon spin coating. Accordingly, the concentration of the dispensed PDMS precursor is used here primarily as a proxy for the resulting SiO_*x*_ thickness.

**Table 1:** Nominal SiO_*x*_ thickness, hydrogen permeability through SiO_*x*_ and hydrogen diffusivity in the aqueous electrolyte for all the investigated electrode samples

Electrode sample	*l*_SiO_*x*__ (nm)	*P*_H_2_,SiO_*x*__ (10^−9^ cm^2^ s^−1^)	*D*_H_2_,aq_ (10^−5^ cm^2^ s^−1^)
50 nm-Pt|Ti	—	—	4.1
[5 mg mL^−1^ PDMS]-SiO_*x*_|50 nm-Pt|Ti	2.3	6.0	3.8
[10 mg mL^−1^ PDMS]-SiO_*x*_|50 nm-Pt|Ti	7.0	7.8	4.5
[20 mg mL^−1^ PDMS]-SiO_*x*_|50 nm-Pt|Ti	8.2	8.2	4.0
edep-Pt|Ti	—	—	4.2
[5 mg mL^−1^ PDMS]-SiO_*x*_ |edep-Pt|Ti	2.7	7.6	4.1
[10 mg mL^−1^ PDMS]-SiO_*x*_ |edep-Pt|Ti	7.4	5.6	4.3
[20 mg mL^−1^ PDMS]-SiO_*x*_ |edep-Pt|Ti	8.1	7.8	2.9

**Fig. 4:**
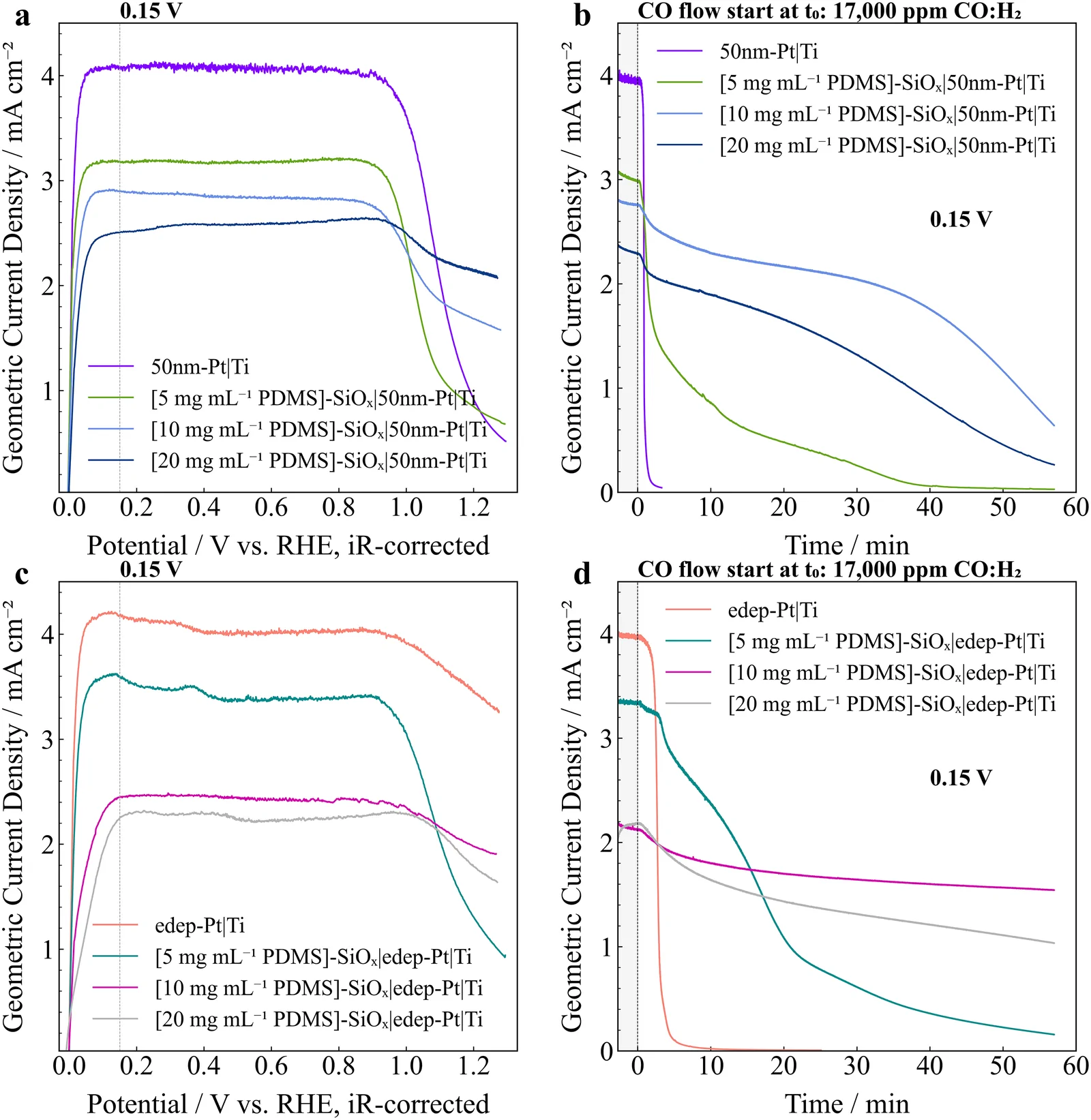
Thickness-dependent HOR activity and CO tolerance of PDMS-derived SiO_*x*_ coatings on 50 nm- and edep-Pt|Ti electrodes. (a) H_2_-saturated, *iR*-corrected linear sweep voltammograms (LSVs) at 2500 rpm and 10 mV s^−1^ for uncoated 50 nm-Pt|Ti and SiO_*x*_-coated 50 nm-Pt|Ti prepared from 5, 10, and 20 mg mL^−1^ PDMS precursor solutions. (b) Corresponding CO-poisoning HOR chronoamperometry at 0.15 V *vs.* RHE (2500 rpm): electrodes are operated in pure H_2_ for 3 min, then CO is introduced at *t*_0_ to obtain 17 000 ppm CO in H_2_. (c) H_2_-saturated, *iR*-corrected LSVs at 2500 rpm and 10 mV s^−1^ for uncoated edep-Pt|Ti and SiO_*x*_-coated edep-Pt|Ti prepared from 5, 10, and 20 mg mL^−1^ PDMS precursor solutions. (d) Corresponding CO-poisoning HOR chronoamperometry at 0.15 V *vs.* RHE (2500 rpm) using the same CO-switch protocol as in (b). Electrolyte for all measurements: 0.1 M HClO_4_ (pH = 1.1) under ambient conditions.

We next quantified how the SiO_*x*_ overlayer impacts HOR activity and CO tolerance. To assess HOR activity, we recorded linear-sweep voltammograms (Fig. 4a and c) in pure H_2_-saturated 0.1 M HClO_4_ using an RDE (2500 rpm, 10 mV s^−1^). The limiting current density of the bare 50 nm-Pt|Ti and edep-Pt|Ti electrodes is ∼4 mA cm^−2^, consistent with Levich-limited H_2_ transport. The corresponding apparent dissolved-H_2_ diffusivity (Fig. S7) is in the order of 4 × 10^−5^ cm^2^ s^−1^ (Table 1), in good agreement with the literature values reported for H_2_ diffusion in water at 25 °C.^61^ Although SEM reveals discontinuous Pt coverage on the edep-Pt|Ti electrode, the estimated hydrodynamic diffusion-layer thickness at 2500 rpm is approximately 16 µm,^62,63^ substantially larger than the characteristic spacing between the Pt domains. The overlapping diffusion fields allow H_2_ transported toward Pt-free regions of the disk to diffuse laterally to nearby Pt domains. Because the HOR kinetics on Pt are sufficiently rapid, the distributed Pt domains act as an effective reactant sink across the full geometric disk area. Thus, the geometric Levich plateau remains essentially unchanged despite differences in Pt morphology, coverage, and ECSA. Because acidic HOR on Pt rapidly becomes mass-transport limited,^64^ and the coating introduces additional transport resistance, a reliable kinetic current extraction is not performed in this work.

In Fig. 4a, upon introduction of the SiO_*x*_ overlayer, the HOR limiting current density for 50 nm-Pt|Ti decreases from the uncoated value to 3.2 mA cm^−2^ for the 5 mg mL^−1^ PDMS-derived coating, 2.8 mA cm^−2^ for 10 mg mL^−1^, and 2.5 mA cm^−2^ for 20 mg mL^−1^. Consistent with this trend, IRRAS indicates nominal SiO_*x*_ thicknesses of 2.3, 7.0, and 8.2 nm for these respective coatings. Similarly, in Fig. 4c for edep-Pt|Ti, the limiting current density decreases to 3.4, 2.4, and 2.2 mA cm^−2^ for the 5, 10, and 20 mg mL^−1^ PDMS-derived SiO_*x*_ coatings, respectively, corresponding to the estimated nominal SiO_*x*_ thicknesses of 2.7, 7.4, and 8.1 nm. For all voltammograms in Fig. 4a and c, as the potential is swept to more positive values, the HOR current decreases because the Pt surface progressively oxidizes to PtO_*x*_. PtO_*x*_ is much less active for the HOR than metallic Pt, and its formation blocks or replaces metallic Pt surface sites, effectively reducing the number of Pt active sites available for H adsorption and H_2_ oxidation.^65^

Taken together, these results establish a clear structure–property relationship in which increasing SiO_*x*_ amount on surface-nominal thickness suppresses the HOR limiting current, consistent with increased mass-transport/permeation resistance for H_2_ through the overlayer and/or a reduced fraction of accessible Pt active sites. Notably, at a given PDMS precursor concentration, the nominal SiO_*x*_ thicknesses estimated for the 50 nm-Pt|Ti and edep-Pt|Ti electrodes are very similar (2.3 *vs*. 2.7 nm, 7.0 *vs*. 7.4 nm, and 8.2 *vs*. 8.1 nm for 5, 10, and 20 mg mL^−1^ PDMS, respectively), and the corresponding HOR limiting currents are likewise closely comparable (Fig. 4). This indicates that the HOR response is governed primarily by the properties of the SiO_*x*_ overlayer on both underlying Pt morphologies.

Based on prior analyses of transport through ultrathin overlayers,^66,67^ we analyzed the HOR limiting currents using a Koutecky–Levich-type framework to separate the two mass-transport contributions governing the measured response (details in SI: Section S4 and Fig. S8, S9). Specifically, the overall limiting current was treated as a resistances-in-series problem, in which H_2_ must first diffuse through the hydrodynamic diffusion boundary layer (DBL) in the electrolyte and then permeate through the SiO_*x*_ overlayer before reaching the Pt surface:1
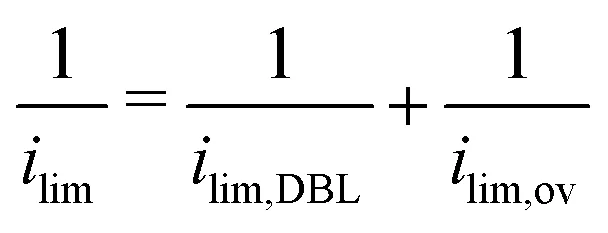


Here, *i*_lim,DBL_ corresponds to the electrolyte-side transport contribution and is governed by H_2_ diffusion in the aqueous phase, from which we extract the dissolved-H_2_ diffusivity, *D*_H_2_,aq_ (10^−5^ cm^2^ s^−1^). In contrast, *i*_lim,ov_ captures the additional transport limitation imposed by the silica overlayer and is used to determine the effective H_2_ permeability through the overlayer, *P*_H_2_,SiO_*x*__ (10^−9^ cm^2^ s^−1^). This treatment, therefore, allows us to decouple bulk electrolyte transport from coating-induced transport resistance and to quantify the transport effect associated with the SiO_*x*_ barrier. The resulting values fall in a relatively narrow band of *P*_H_2_,SiO_*x*__ = (6–8) × 10^−9^ cm^2^ s^−1^ for coating thicknesses of ∼2.5–8 nm (Table 1). The measured values are higher than the recently reported value of (3.8 ± 0.4) × 10^−10^ cm^2^ s^−1^ for a 10 nm undoped ALD SiO_2_ membrane.^68^ This difference may be associated with the smaller nominal thickness of the tested SiO_*x*_ layers, as well as differences in film composition and substrate morphology. Apart from *P*_H_2__, the derived diffusivity of dissolved H_2_ in the aqueous electrolyte is *D*_H_2_,aq_= (3–4.5) × 10^−5^ cm^2^ s^−1^, consistent with literature values.^61,69^ Thus, the SiO_*x*_ layer imposes an H_2_ transport coefficient that is approximately 4 orders of magnitude smaller than H_2_ diffusion in the bulk aqueous electrolyte.

After the HOR measurements in a pure H_2_ atmosphere, we evaluated CO tolerance by potentiostatic HOR (chronoamperometry) at + 0.15 V *vs.* RHE, *i.e.*, less anodic than the CO-stripping region while operating at the mass-transport-limited HOR plateau at 2500 rpm (similarly to the HOR LSV tests). Following an initial stabilization period in pure H_2_, CO was introduced at *t*_0_ = 0 s to reach 17 000 ppm CO in the H_2_ gas feed (Fig. 4b and d). The uncoated 50 nm-Pt|Ti electrode deactivates rapidly, approaching complete poisoning within minutes (Fig. 4b). In contrast, all SiO_*x*_-coated 50 nm-Pt|Ti electrodes exhibit a markedly slower current decay, demonstrating that the overlayer hinders CO access to the underlying Pt surface. Together with the IRRAS-derived thicknesses and the HOR voltammograms in a pure H_2_ atmosphere, a structure–property relationship emerges again. The thinnest coating, derived from 5 mg mL^−1^ PDMS (∼2.3 nm SiO_*x*_), provides only partial protection against CO and deactivates faster than the thicker coatings, indicating that such a thin film is insufficient to effectively block CO transport towards Pt. Increasing the coating thickness to the intermediate regime, namely the 10 mg mL^−1^ PDMS-derived film with a thickness of ∼7.0 nm, gives the best balance between HOR activity and poisoning resistance: although the initial HOR limiting current in a pure H_2_ atmosphere is reduced relative to the thinnest coating, this sample shows the highest current retention over 1 h of CO exposure.

In contrast, the thickest film, obtained from 20 mg mL^−1^ PDMS and reaching ∼8.2 nm, leads to the largest HOR activity loss but does not outperform the 10 mg mL^−1^ coating in CO tolerance. The FT-IRRA spectra in Fig. 3 show that the 20 mg mL^−1^ sample does not form a fully converted amorphous SiO_*x*_ layer, but instead retains substantial disorder and residual carbonaceous species. This observation demonstrates that a thicker but lower-quality film suppresses the initial HOR activity in a pure H_2_ atmosphere more strongly, but it does not provide improved CO tolerance. Eventually, all the coated 50 nm-Pt|Ti electrodes show a pronounced loss of CO tolerance after ∼40 min, suggesting a time-dependent failure mode of the SiO_*x*_ barrier, for example, through progressive loss of barrier integrity or the emergence of permeation pathways that allow CO to access and poison Pt sites more readily.

A related trend is observed for the corresponding coatings on edep-Pt|Ti (Fig. 4d), where the overall CO tolerance is clearly improved relative to the coated 50 nm-Pt|Ti samples. In particular, after an initially slow deactivation, the 10 mg mL^−1^ PDMS SiO_*x*_|edep-Pt|Ti electrode retains the highest fraction of its initial HOR current density in a pure H_2_ atmosphere after 1 h, again identifying the intermediate-thickness coating as the optimum. Since the IRRAS-derived nominal SiO_*x*_ thicknesses are very similar on the two substrates at a given PDMS loading, the improved CO tolerance on edep-Pt|Ti is not attributed simply to a difference in coating thickness. Rather, these results suggest that substrate-dependent factors, such as differences in the underlying Pt morphology and/or in the overlayer–substrate interface, also influence how effectively the SiO_*x*_ film is formed and how it delays CO poisoning.

Nevertheless, the same qualitative trend is observed on both substrates: the thinnest film provides insufficient protection, the intermediate thickness film gives the best compromise between HOR activity and CO tolerance, and the thickest film does not deliver an additional benefit since it also does not form a fully converted amorphous SiO_*x*_ layer. Extending the UV–ozone treatment to 6 h for an additional 10 mg mL^−1^ PDMS-derived sample did not completely remove the weak residual methyl-related IRRAS feature (Fig. S10). Thus, prolonged treatment alone does not guarantee complete conversion, particularly for thicker precursor layers like in the 20 mg mL^−1^ sample.

To further support these conclusions, reproducibility tests were carried out to validate the observed trends of the CA traces (Fig. S11). For the coated 50 nm-Pt|Ti samples, the relative standard deviation was 1% for the 5 mg mL^−1^ PDMS-derived SiO_*x*_ sample and 9% for both the 10 and 20 mg mL^−1^ samples. For the coated edep-Pt|Ti samples, the corresponding relative standard deviations were approximately 5% for the 5 mg mL^−1^ sample and approximately 10% for the 10 mg mL^−1^ sample. The 20 mg mL^−1^ edep-Pt|Ti sample was not investigated further, since the corresponding coating on 50 nm-Pt|Ti already indicated non-ideal overlayer quality. These deviations should be considered when comparing individual traces and arise primarily from variability in the spin-coating process, which is not inherently sufficiently controlled and therefore introduces sample-to-sample differences in SiO_*x*_ formation. However, this variability level does not alter the overall interpretation of the data and we can conclude that the CO-poisoning response is governed by a combined structure–property relationship in which the thickness, the chemical/structural quality as well as the underlying Pt morphology of the SiO_*x*_ overlayer control the poisoning resistance.

Expanding on CO permeation through the SiO_*x*_ overlayer, the early-time chronoamperometric response can be related to directly accessible Pt regions. By attributing the entire initial rapid current decrease to directly accessible Pt and normalizing it to the initial bare-Pt current, we estimate upper-bound equivalent exposed-Pt fractions of approximately 36%, 7%, and 6% for the 5, 10, and 20 mg mL^−1^ coatings on planar 50 nm-Pt|Ti (Fig. 4b), respectively. Applying the same analysis to the electrodeposited Pt electrodes (Fig. 4d) yields conservative values of approximately 13%, 6%, and 9%. The initial decay may also include contributions from coating damage at the disk edge during mounting.

To elaborate further on the CO mitigation of the SiO_*x*_ overalyer, the chronoamperometric results are further supported by CO-stripping measurements (details in SI: Section S7 and Fig. S12, S13). For the uncoated edep-Pt|Ti electrode, both the CO-derived ECSA and the independently determined *H*_upd_-derived ECSA were 2.8 cm^2^, confirming the consistency of the two measurements. Following deposition of the [10 mg mL^−1^ PDMS]-SiO_*x*_ overlayer, the *H*_upd_-derived ECSA decreased to 1.9 cm^2^, whereas the CO-derived ECSA decreased more strongly to 0.5 cm^2^. These values correspond to reductions of approximately 32% and 82%, respectively, indicating that the decrease in CO adsorption capacity cannot be explained solely by a general loss of electrochemically accessible Pt.

Consistently, the coated electrode retained approximately 72% of its initial HOR activity in a pure H_2_ atmosphere after 57 min of continuous CO exposure, whereas the uncoated electrode lost essentially all activity within approximately 8 min. These combined results support preferential restriction of CO access and accumulation relative to proton and H_2_ transport through the SiO_*x*_ overlayer. This suggests a transport mechanism through interstitial free-volume sites in silica, resulting in substantially lower CO permeation because of its larger molecular size. Definitive confirmation of this mechanism, however, would require a more detailed investigation of the nanometric pore-size distribution before the hypothesis can be generalized. As shown in Fig. S12 and S13, the main CO-stripping peak shifted only slightly from 0.74 to 0.76 V *vs.* RHE, whereas the pre-stripping feature at approximately 0.58 V disappeared after coating. This feature may reflect a weakly adsorbed or kinetically less stable CO, but can also depend on CO coverage, Pt surface structure, and the nucleation of oxygenated species at step or defect sites that take part in CO oxidation.^70^ These observations alone are therefore not taken as direct evidence of a change in CO binding energy upon SiO_*x*_ coating.

### 3.3. Stability and HOR energy demand of SiO_*x*_-coated Pt under CO

Industrially relevant H_2_ feedstock streams (*e.g.*, reformate/shifted gas from hydrocarbon reforming or fuel processing) typically contain CO along with other impurities such as CO_2_, CH_4_, H_2_ O, N_2_, and trace sulfur species (*e.g.*, H_2_S, COS, and mercaptans).^71^ The CO level in such impure hydrogen streams is commonly in the 10^3^–10^4^ ppm(v) range (approximately 0.1–2 vol%), depending on the extent of water–gas shift and downstream cleanup.^72,73^ In large-scale steam methane reforming (SMR) plants, CO concentrations are typically on the order of ∼8–10 vol% upstream of the shift reactors and decrease to ∼0.4–0.8 vol% (4000–8000 ppm) after the low-temperature shift stage, prior to the final purification of the hydrogen product.^71^

**Fig. 5:**
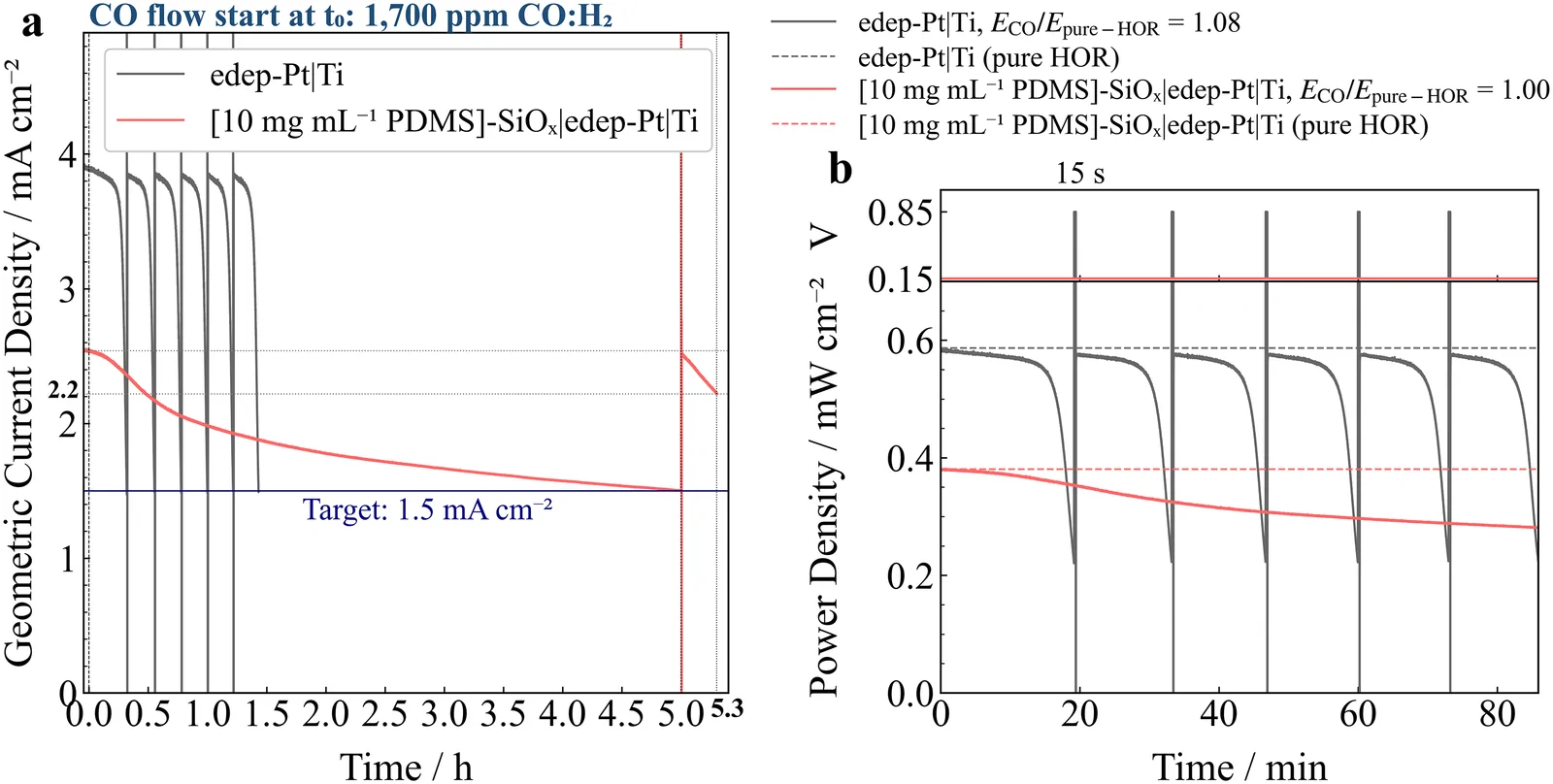
CO poisoning–stripping stability analysis during potentiostatic HOR at 0.15 V *vs*. RHE (2500 rpm) in 1700 ppm CO:H_2_. (a) Geometric current density during continuous CO exposure. Once the current decays to the defined threshold of 1.5 mA cm^−2^, a 15 s pulse to 0.85 V *vs*. RHE is applied to oxidatively remove adsorbed CO, after which the potential is returned to 0.15 V while maintaining the CO-containing feed. (b) Time-resolved potential trace (top) and the corresponding power density, over the common post-*t*_0_ comparison window of 86 min. Dashed horizontal lines indicate the pure HOR reference power density estimated from the average pre-CO current density. Electrolyte for all measurements: 0.1 M HClO_4_ (pH = 1.1) under ambient conditions.

To quantify the characteristic poisoning time under a defined current loss, we performed pulsed CO poisoning–stripping during potentiostatic HOR at 0.15 V *vs*. RHE (2500 rpm) upon saturating the electrolyte with 1700 ppm CO/H_2_, an industrially relevant impurity level. We set a practical threshold at 1.5 mA cm^−2^ (40% loss) and once reached, a short 15 s stripping pulse at 0.85 V was applied to oxidatively remove adsorbed CO (to CO_2_), after which the potential was set again to 0.15 V while maintaining continuous CO flow. As shown in Fig. 5a, the uncoated edep-Pt|Ti electrode reaches the threshold after ∼19 min in the first cycle, whereas subsequent cycles proceed with reproducible poisoning intervals of ∼14 min. The longer first cycle indicates that ∼5 min are required for the dissolved CO concentration to approach its steady-state value under the chosen gas-feed and hydrodynamic conditions. After this initial equilibration period, the poisoning kinetics of the uncoated electrode become highly reproducible.

By contrast, the 10 mg mL^−1^ SiO_*x*_|edep-Pt|Ti electrode requires ∼5 h to decay to the same current threshold. Then, a single 15 s stripping pulse restores the HOR current essentially to its initial value upon returning to 0.15 V, despite continued exposure to 1700 ppm CO/H_2_. The more rapid current decrease following the stripping pulse does not indicate accelerated degradation. The electrolyte and overlayer were already equilibrated with CO at this point (*t* = 5 h), causing faster re-poisoning compared to the initial transient (from *t* = 0) that includes the time required for CO saturation. Moreover, post-mortem LEIS (Fig. S14) after 5 h of operation shows only O and Si features without detectable Pt, indicating that no overlayer delamination occurred.

The two 1700 ppm CO-poisoning transients were also fitted using a custom two-population Johnson–Mehl–Avrami–Kolmogorov (JMAK) model in which Pt deactivation is driven by cumulative CO exposure,^74^ while the finite time required to establish dissolved CO in the electrolyte was incorporated through a fixed mixing term (*τ*_m_ = 300 s; details are provided in the SI: Section S9 and Fig. S15, S16 and Table S1). The fit is interpreted phenomenologically as a description of the apparent current-decay behavior. Relative to uncoated edep-Pt|Ti, the coated electrode exhibits a larger nonzero long-time current and a substantially prolonged slow-poisoning timescale. The uncoated electrode shows a faster-than-exponential, cooperative-like apparent decay, whereas the coated electrode approaches an apparent first-order decay.

Because the coated and uncoated edep-Pt|Ti electrodes show different Pt poisoning kinetics before reaching the HOR current-loss threshold, comparing their energy demand at identical elapsed time would not correspond to equal H_2_ throughput. We therefore used an equal-charge analysis to quantify the energetic penalty associated with CO poisoning.

First, the total post-*t*_0_ charge passed by the uncoated edep-Pt|Ti over the full poisoning experiment recorded (86 min) was taken as the target charge. Then, for the coated edep-Pt|Ti, the measured CO-poisoning trace was integrated until the same charge had been delivered, *i.e.* until t* satisfied:2
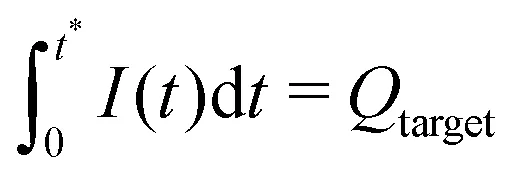


The corresponding energy demand of both electrodes under CO was calculated as:3
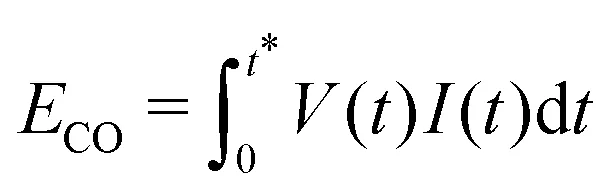


In order to evaluate the energy demand of the initial HOR in a pure H_2_ atmosphere, the average current during the first 3 min before CO introduction (−3 to 0 min) was taken as the electrode-specific baseline current, while the potential was fixed at the applied value of 0.15 V *vs*. RHE for both electrodes. The pure HOR energy demand for the same target charge throughput is therefore:

4 *E*_pure-HOR_ = 0.15*Q*_target_

All time integrals were evaluated numerically from the measured current (Fig. 5a) and potential traces (top panel of Fig. 5b). For both uncoated and coated electrodes, this equal-charge comparison isolates the CO-induced energetic penalty from differences in poisoning lifetime and keeps the HOR throughput fixed. Therefore, the *E*_CO_/*E*_pure-HOR_ ratio directly expresses the additional energy demand caused by CO poisoning at equal charge throughput compared to the pure HOR operation for each electrode. Since no oxidative pulses were performed with the silica coated edep-Pt|Ti for the target charge duration, the ratio is 1 and it denotes no excess energy demand relative to pure HOR, due to the same applied potential at 0.15 V for both CO poisoning and pure HOR operations. On the other hand, the ratio of the uncoated edep-Pt|Ti is 1.08, corresponding to an additional 8% energy demand penalty which arises from the oxidative potential pulsing at 0.85 V upon CO exposure. Notably, the periodic CO-stripping protocol applied already operates close to its practical optimum, as the associated energy loss is only about 8%.

To visualize how this energetic penalty develops in time, Fig. 5b additionally plots the potential trace (top panel) and the resulting power density (bottom panel), calculated as *p*(*t*) = *V*(*t*)*j*(*t*), where *j*(*t*) is the geometric HOR current density. For this comparison, both datasets were evaluated over the same post-*t*_0_ time window (86 min), defined by the full duration of the uncoated edep-Pt|Ti experiment. The dashed horizontal lines in Fig. 5b represent the corresponding pure HOR reference power density, *p*_pure-HOR_ = 0.15*j̄*_HOR_, with the average current density (*j̄*_HOR_) obtained from the −3 to 0 min baseline before CO exposure. The uncoated edep-Pt|Ti shows repeated collapses in power density as CO accumulates, followed by transient recovery during each stripping pulse, yielding a measurable energy penalty relative to pure HOR. The time-averaged (86 min post *t*_0_) HOR power density for the uncoated edep-Pt|Ti is 0.58 mW cm^−2^, a 1.8 times higher value than the silica-coated Pt delivering an average of 0.32 mW cm^−2^.

In contrast, the 10 mg mL^−1^ SiO_*x*_|edep-Pt|Ti retains a much more gradual decay in power density over the same comparison window and remains substantially closer to its own pure-HOR reference, consistent with strongly mitigated CO-induced energy losses. From a practical standpoint, this steadier output may be preferable to the higher instantaneous power required through periodic recovery pulses, because real galvanostatic operation in PEM electrochemical systems can complicate operation (no fine control over the anode potential) and accelerate degradation, and durability remains a central challenge for electrochemical hydrogen compressors.^9,75^

**Fig. 6:**
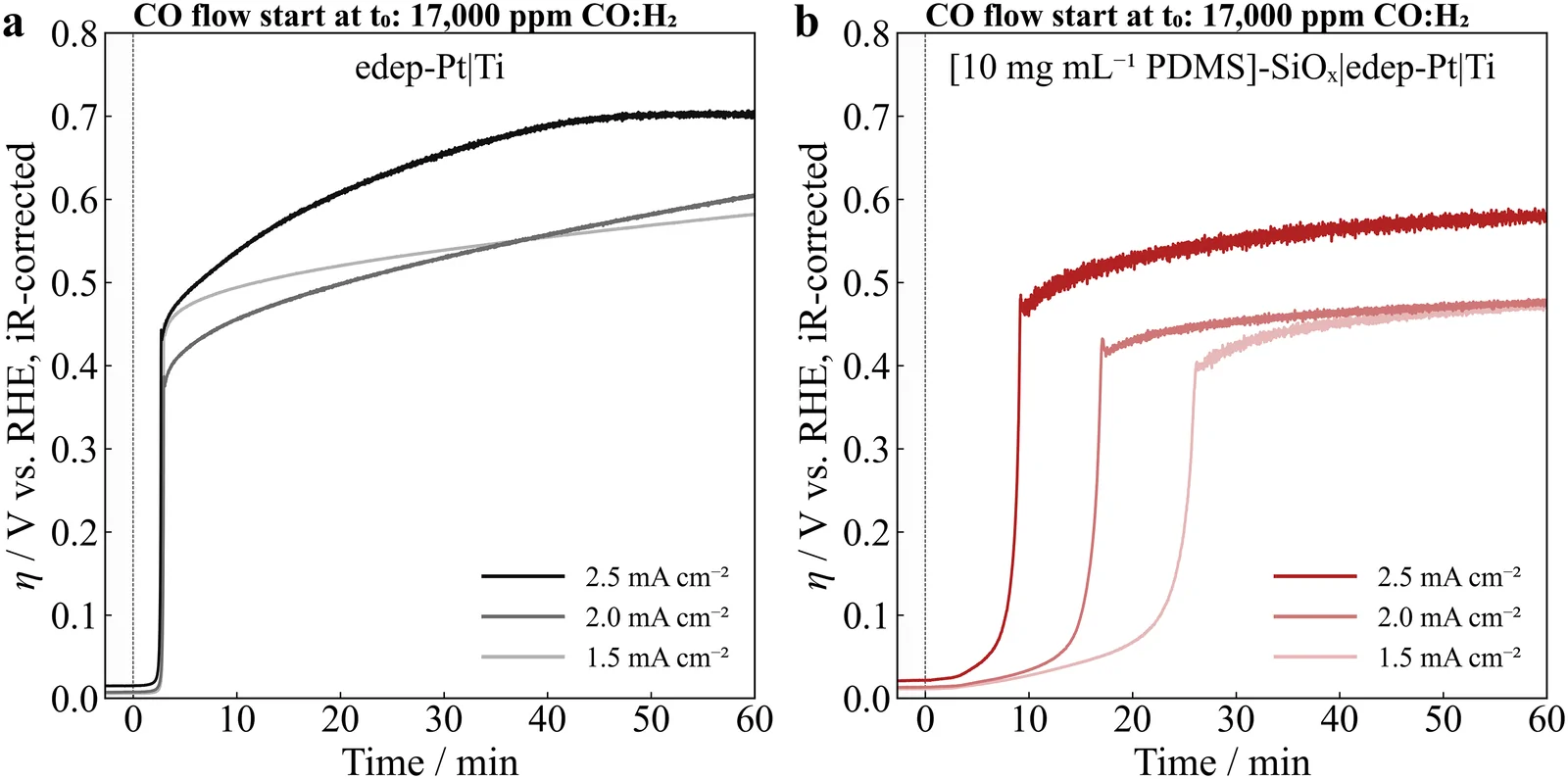
Chronopotentiometric HOR operation in 17 000 ppm CO in H_2_ at 2500 rpm. Shown are *iR*-corrected overpotentials, *η*, at applied current densities of 1.5, 2.0, and 2.5 mA cm^−2^. The dashed vertical line marks the start of CO dosing at *t*_0_: (a) edep-Pt|Ti; and (b) [10 mg mL^−1^ PDMS]-SiO_*x*_|edep-Pt|Ti. Electrolyte for all measurements: 0.1 M HClO_4_ (pH = 1.1) under ambient conditions.

To further investigate the energetic penalty now imposed again by severe CO contamination, chronopotentiometry (CP) was performed at 17 000 ppm CO in H_2_ and 2500 rpm at applied current densities of 1.5, 2.0, and 2.5 mA cm^−2^, a range below the HOR limiting current for both electrodes tested (Fig. 6). For uncoated edep-Pt|Ti (Fig. 6a), switching to the CO-containing feed at *t*_0_ causes an immediate rise in the overpotential, consistent with rapid CO adsorption on available Pt sites. Then, the potential increases continuously with time, reaching at the end of the experiment approximately 0.58, 0.60, and 0.70 V *vs.* RHE for 1.5, 2.0, and 2.5 mA cm^−2^, respectively. This behavior indicates ongoing poisoning coverage under constant CO supply, such that progressively higher anodic overpotential is required to sustain the applied current.

In comparison, [10 mg mL^−1^ PDMS]-SiO_*x*_|edep-Pt|Ti (Fig. 6b) introduces a pronounced delay period post *t*_0_, in which the electrode operates at near-baseline overpotential as it does in the first 3 min of pure HOR (pre-*t*_0_), less than 0.05 V, despite CO in the feed, indicating strongly suppressed CO access to the Pt surface. The duration of this period decreases with increasing current density (approximately 25–27 min at 1.5 mA cm^−2^, 15–17 min at 2.0 mA cm^−2^, and 8–10 min at 2.5 mA cm^−2^), after which *η* increases to a new steady operating level. Even after this transition, the coated electrode exhibits only a modest drift in *η* over the remainder of the experiment (ending at 0.47, 0.48, and 0.58 V for 1.5, 2.0, and 2.5 mA cm^−2^, respectively). These results show that the SiO_*x*_ layer both delays the onset of CO poisoning and reduces the sustained overpotential required to maintain a given applied current density compared to uncoated Pt.

To compare the cumulative anodic energy demand under these constant-current conditions, we evaluated the energy ratio *E*_CO_/*E*_pure-HOR_ from the *iR*-corrected CP traces. For each experiment, the pure HOR reference overpotential, *η̄*_pure-HOR_, was taken as the time-weighted average overpotential during the 3 min preceding CO introduction (−180 to 0 s). The corresponding pure HOR energy demand over the same post-*t*_0_ analysis window was then estimated as:

5 *E*_pure-HOR_ = *Iη̄*_pure-HOR_*t*_f_

where *I* is the applied current and *t*_f_ = 3420 s is the post-CO integration window. The actual cumulative energy demand under CO poisoning was then obtained from:6
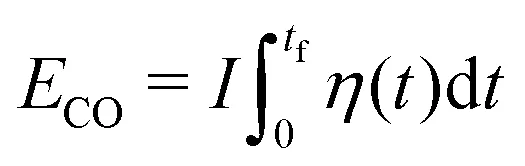


**Fig. 7:**
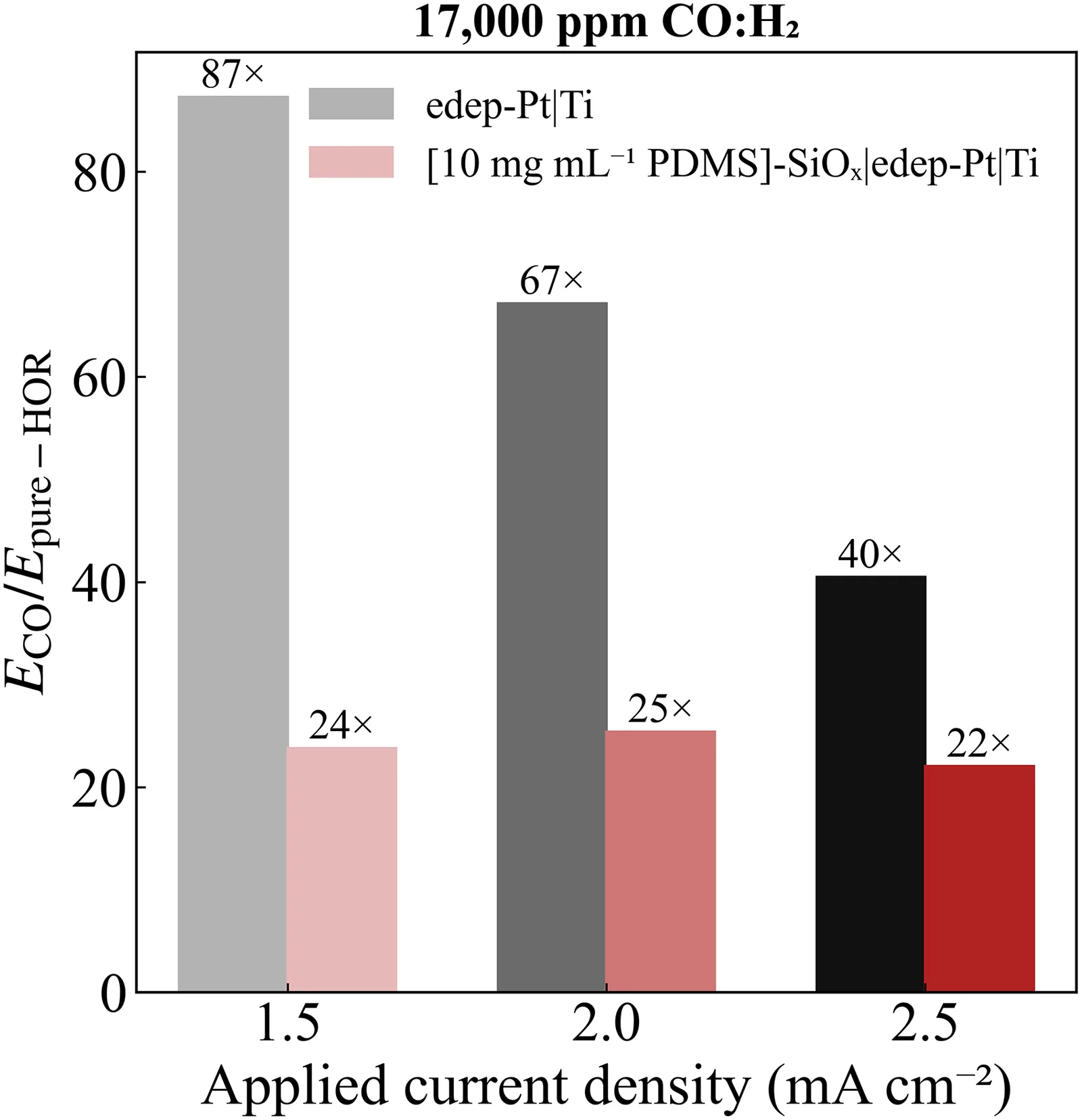
Energy demand ratio *E*_CO_/*E*_pure-HOR_ derived from the chronopotentiometric traces in Fig. 6, with *E*_CO_ and *E*_pure-HOR_, both evaluated over the same post-*t*_0_ duration of 57 min. Electrolyte for all measurements: 0.1 M HClO_4_ (pH = 1.1) under ambient conditions.

The resulting ratio *E*_CO_/*E*_pure-HOR_ is plotted in bars in Fig. 7, and for each electrode, it quantifies how many times larger the anodic energy demand becomes under CO-contaminated operation than under pure HOR operation at the same applied current density and over the same duration. For bare edep-Pt|Ti, *E*_CO_/*E*_pure-HOR_ decreases from 87 at 1.5 mA cm^−2^ to 67 at 2.0 mA cm^−2^ and 40 at 2.5 mA cm^−2^. This downward trend should not be interpreted as improved CO tolerance at higher current density. Instead, it reflects normalization to a progressively larger pure HOR *η* baseline. Under CO, the electrode is rapidly driven into a roughly similar high-overpotential regime at all three currents, so the relative energy ratio is largest where the pure HOR reference energy is smallest, *i.e.* at 1.5 mA cm^−2^.

In comparison, the coated electrode displays an almost applied current-independent ratio, with *E*_CO_/*E*_pure-HOR_= 24, 25, and 22 at 1.5, 2.0, and 2.5 mA cm^−2^, respectively. This constant behavior indicates that the SiO_*x*_ overlayer suppresses the CO-induced increase in overpotential in a proportional manner across the applied current density range. The coating reduces the cumulative anodic energy demand under CO by roughly a factor of 3.6, 2.7, and 1.8 relative to uncoated edep-Pt|Ti at 1.5, 2.0, and 2.5 mA cm^−2^ (∼3 times for the total range), respectively, scaling with the CO delay period. This result demonstrates that despite the pure HOR activity loss due to the presence of the SiO_*x*_ overlayer (CA traces), operating with optimal SiO_*x*_ coatings is still more energetically favorable than uncoated Pt operating at the HOR benchmark limiting current.

## 4. Conclusions

Ultrathin PDMS-derived SiO_*x*_ overlayers provide a tunable strategy for improving the CO tolerance of Pt HOR anodes. Their performance depends on overlayer thickness, coating quality, and substrate morphology. Among the investigated electrodes, the coated edep-Pt|Ti electrode with a nominal overlayer thickness of approximately 7 nm provided the best balance between HOR activity and CO resistance. It sustained operation for several hours under 1700 ppm CO, whereas uncoated Pt became fully poisoned within 10 min. In addition, regeneration by brief oxidative pulses preserved the coating integrity. CO-stripping measurements further showed that the overlayer suppresses CO adsorption more strongly than it reduces the electrochemically accessible Pt area. At 17 000 ppm CO under constant-current operation, the coating also reduced the cumulative anodic energy demand by up to 3.6-fold relative to uncoated Pt. These findings establish ultrathin SiO_*x*_ overlayers as a promising CO-mitigation strategy because they substantially prolong operation before poisoning while maintaining a high HOR current density. A key challenge will be their translation to gas-diffusion electrodes (GDEs). The faster gas-phase reactant supply and coating-induced changes in GDE-layer wettability may alter the relative H_2_ and CO fluxes at the Pt surface. Successful scale-up will therefore require selective coating of the catalyst particles while minimizing changes to the support that could cause flooding or pore blockage. Selective ALD offers a promising route for conformal coating of GDEs, and preliminary measurements on an ALD-coated edep-Pt|Ti electrode showed HOR activity and CO tolerance comparable to those of the wet-chemically prepared coating used in this work (Fig. S17).

## Conflicts of interest

The authors declare no conflict of interest.

## Supplementary Material

EY-004-D6EY00101G-s001

## Data Availability

The raw and processed data supporting this study will be made publicly available on Zenodo at https://doi.org/10.5281/zenodo.22679630. All data presented in the figures are derived from this dataset. Supplementary information (SI) contains additional surface characterization, Pt ECSA measurements, SiO_*x*_ thickness and H_2_ permeability analyses, replicate CO-poisoning data, CO-stripping and LEIS measurements, CO-poisoning kinetic modeling, and control experiments. See DOI: https://doi.org/10.1039/d6ey00101g.

## References

[cit1] Climate change 2022: mitigation of climate change, ed., P. R. Shukla, J. Skea, A. R. Reisinger, IPCC, Geneva, 2022, ISBN: 978-92-9169-160-9

[cit2] International Energy Agency. The Future of Hydrogen: Seizing Today's Opportunities. Tech. rep. Paris: International Energy Agency, 2019

[cit3] Mekonnin A. S., Wacawiak K., Humayun M., Zhang S., Ullah H. (2025). Hydrogen Storage Technology, and Its Challenges: A Review. Catalysts.

[cit4] Rhandi M., Trégaro M., Druart F., Deseure J., Chatenet M. (2020). Electrochemical hydrogen compression and purification versus competing technologies: Part I. Pros and cons. Chin. J. Catal..

[cit5] Durmus G. N. B., Colpan C. O., Devrim Y. (2021). A review on the development of the electrochemical hydrogen compressors. J. Power Sources.

[cit6] Bagacki R., Reinhardt M., Schlatmann R., Calnan S., Van De Krol R., Browne M. P. (2025). Electrochemical hydrogen pumps: a researcher's guide and review. Chem. Commun..

[cit7] Marciu D., Kova A., Firak M. (2022). Electrochemical hydrogen compressor: Recent progress and challenges. Int. J. Hydrogen Energy.

[cit8] Chouhan A., Bahar B., Prasad A. K. (2020). Effect of Back-Diffusion on the Performance of an Electrochemical Hydrogen Compressor. Int. J. Hydrogen Energy.

[cit9] Pivac I., Stoilova Pavasovi A., Barbir F. (2024). Recent advances and perspectives in diagnostics and degradation of electrochemical hydrogen compressors. Int. J. Hydrogen Energy.

[cit10] Zängler W. , Semrau N., Wessling M. and Keller R., Effect of Natural Gas Impurities on Electrochemical Hydrogen Compression and Strategies for Mitigation, 2025 10.2139/ssrn.5081106

[cit11] Ketter F., Jha S., Mürtz S. D., Palkovits R. (2025). Integration of formic acid dehydrogenation with electrochemical pumping for the generation of clean hydrogen. Sustainable Energy Fuels.

[cit12] Perry K. A., Eisman G. A., Benicewicz B. C. (2008). Electrochemical Hydrogen Pumping Using a High-Temperature Polybenzimidazole (PBI) Membrane. J. Power Sources.

[cit13] Bouwman P. (2014). Electrochemical Hydrogen Compression (EHC) Solutions for Hydrogen Infrastructure. Fuel Cells Bull..

[cit14] Valdés-López V. F., Mason T., Shearing P. R., Brett D. J. (2020). Carbon monoxide poisoning and mitigation strategies for polymer electrolyte membrane fuel cells A review. Prog. Energy Combust. Sci..

[cit15] Das S. K., Reis A., Berry K. (2009). Experimental evaluation of CO poisoning on the performance of a high temperature proton exchange membrane fuel cell. J. Power Sources.

[cit16] Jackson C., Raymakers L., Mulder M., Kucernak A. (2020). Assessing electrocatalyst hydrogen activity and CO tolerance: Comparison of performance obtained using the high mass transport floating electrode technique and in electrochemical hydrogen pumps. Appl. Catal., B.

[cit17] Gardner C., Ternan M. (2007). Electrochemical separation of hydrogen from reformate using PEM fuel cell technology. J. Power Sources.

[cit18] Jha S., Mebrahtu C., Ott F., Jiang C., Hartmann H., Palkovits R. (2026). An Efficient PdPt/rGO Catalyst for Hydrogen Electrolysis in Acidic Medium: Structure-Activity Relationships and CO-Tolerance. ChemElectroChem.

[cit19] Brouzgou A., Seretis A., Song S., Shen P. K., Tsiakaras P. (2021). CO tolerance and durability study of PtMe(Me = Ir or Pd) electrocatalysts for H2-PEMFC application. Int. J. Hydrogen Energy.

[cit20] Molochas C., Tsiakaras P. (2021). Carbon Monoxide Tolerant Pt-Based Electrocatalysts for H2-PEMFC Applications: Current Progress and Challenges. Catalysts.

[cit21] Lee S. J., Mukerjee S., Ticianelli E. A., McBreen J. (1999). Electrocatalysis of CO tolerance in hydrogen oxidation reaction in PEM fuel cells. Electrochim. Acta.

[cit22] Qi Z., Kaufman A. (2003). CO-tolerance of low-loaded Pt/Ru anodes for PEM fuel cells. J. Power Sources.

[cit23] Watanabe M., Motoo S. (1975). Electrocatalysis by ad-atoms: Part III. Enhancement of the oxidation of carbon monoxide on platinum by ruthenium ad-atoms. J. Electroanal. Chem. Interfacial Electrochem..

[cit24] Wang M., Pei P., Xu Y. (2024). *et al.*, CO-tolerance behaviors of proton exchange membrane fuel cell stacks with impure hydrogen fuel. Appl. Energy.

[cit25] Wang Y., Wang G., Li G. (2015). *et al.*, Pt–Ru Catalyzed Hydrogen Oxidation in Alkaline Media:Oxophilic Effect or Electronic Effect?. Energy Environ. Sci..

[cit26] Alia S. M., Ha M.-A., Ngo C., Anderson G. C., Ghoshal S., Pylypenko S. (2020). Platinum–Nickel Nanowires with Improved Hydrogen Evolution Performance in Anion Exchange Membrane-Based Electrolysis. ACS Catal..

[cit27] Stamenkovic V. R., Mun B. S., Mayrhofer K. J. J., Ross P. N., Markovic N. M. (2006). Effect of Surface Composition on Electronic Structure, Stability, and Electrocatalytic Properties of Pt-Transition Metal Alloys: Pt-Skin versus Pt-Skeleton Surfaces. J. Am. Chem. Soc..

[cit28] Schmidt T. J., Ross P. N., Markovic N. M. (2002). Temperature dependent surface electrochemistry on Pt single crystals in alkaline electrolytes: Part 2. The hydrogen evolution/oxidation reaction. J. Electroanal. Chem..

[cit29] Rodríguez P., García G., Herrero E., Feliu J. M., Koper M. T. M. (2011). Effect of the Surface Structure of Pt(100) and Pt(110) on the Oxidation of Carbon Monoxide in Alkaline Solution: an FTIR and Electrochemical Study. Electrocatalysis.

[cit30] Feliu J. M., Herrero E. (2024). Pt single crystal surfaces in electrochemistry and electrocatalysis. EES Catal..

[cit31] Katsoukis G., Frei H. (2019). Ultrathin oxide layers for nanoscale integration of molecular light absorbers, catalysts, and complete artificial photosystems. J. Chem. Phys..

[cit32] Leon-Chaparro D., Nguyen M. D., Baeumer C., Mul G., Katsoukis G. (2025). Mechanistic Insights Into Proton and Oxygen Transport Through Ultrathin Amorphous Al_2_O_3_ and Al_2_O_3_–SiO_2_ Electrocatalyst Overlayers. Adv. Mater. Interfaces.

[cit33] Beatty M. E. S., Chen H., Labrador N. Y., Lee B. J., Esposito. D. V. (2018). Structureproperty relationships describing the buried interface between silicon oxide overlayers and electrocatalytic platinum thin films. J. Mater. Chem. A.

[cit34] Worsley M., Smulders V., Mei B. (2022). Controlled Synthesis of Chromium-Oxide-Based Protective Layers on Pt: Influence
of Layer Thickness on Selectivity. Catalysts.

[cit35] Qureshi M., Shinagawa T., Tsiapis N., Takanabe K. (2017). Exclusive Hydrogen Generation by Electrocatalysts Coated with an Amorphous Chromium-Based Layer Achieving Efficient Overall Water Splitting. ACS Sustainable Chem. Eng..

[cit36] Endrdi B., Smulders V., Simic N. (2019). *et al.*, *In situ* formed vanadium-oxide cathode coatings for selective hydrogen production. Appl. Catal., B.

[cit37] Geppert T. N., Bosund M., Putkonen M. (2020). *et al.*, HOR Activity of Pt-TiO_2_-Y at Unconventionally High Potentials Explained: The Influence of SMSI on the Electrochemical Behavior of Pt. J. Electrochem. Soc..

[cit38] Marichy C., Ercolano G., Caputo G. (2016). *et al.*, ALD SnO_2_ protective decoration enhances the durability of a Pt based electrocatalyst. J. Mater. Chem. A.

[cit39] Esposito D. V. (2018). Membrane-Coated Electrocatalysts An Alternative Approach To Achieving Stable and Tunable Electrocatalysis. ACS Catal..

[cit40] Liu Q., Ranocchiari M., Van Bokhoven J. A. (2022). Catalyst overcoating engineering towards high-performance electrocatalysis. Chem. Soc. Rev..

[cit41] Qu J., Urban A. (2020). Potential and pH Dependence of the Buried Interface of Membrane-Coated Electrocatalysts. ACS Appl. Mater. Interfaces.

[cit42] Li M., Saedy S., Fu S., Stellema T., Kortlever R., van Ommen J. R. (2024). Enhancing the durability of Pt nanoparticles for water electrolysis using ultrathin SiO_2_ layers. Catal. Sci. Technol..

[cit43] Jo W. J., Katsoukis G., Frei H. (2020). Ultrathin Amorphous Silica Membrane Enhances Proton Transfer across Solid-to-Solid Interfaces of Stacked Metal Oxide Nanolayers while Blocking Oxygen. Adv. Funct. Mater..

[cit44] Labrador N. Y., Songcuan E. L., De Silva C. (2018). *et al.*, Hydrogen Evolution at the Buried Interface between Pt Thin Films and Silicon Oxide Nanomembranes. ACS Catal..

[cit45] Robinson J. E., Labrador N. Y., Chen H., Sartor B. E., Esposito D. V. (2018). Silicon Oxide-Encapsulated Platinum Thin Films as Highly Active Electrocatalysts for Carbon Monoxide and Methanol Oxidation. ACS Catal..

[cit46] Bhardwaj A. A., Vos J. G., Beatty M. E. S. (2021). *et al.*, Ultrathin Silicon Oxide Overlayers Enable Selective Oxygen Evolution from Acidic and Unbuffered pH-Neutral Seawater. ACS Catal..

[cit47] Harsha S., Sharma R. K., Dierner M. (2024). *et al.*, Dewetting of Pt Nanoparticles Boosts Electrocatalytic Hydrogen Evolution Due to Electronic Metal-Support Interaction. Adv. Funct. Mater..

[cit48] Duarte M., Pilla A., Sieben J., Mayer C. (2006). Platinum particles electrodeposition on carbon substrates. Electrochem. Commun..

[cit49] Pawlus P., Reizer R., Wieczorowski M. (2021). Functional Importance of Surface Texture Parameters. Materials.

[cit50] Cai D., Neyer A., Kuckuk R., Heise H. M. (2010). Raman, mid-infrared, near-infrared and ultravioletvisible spectroscopy of PDMS silicone rubber for characterization of polymer optical waveguide materials. J. Mol. Struct..

[cit51] Bokobza L., Buffeteau T., Desbat B. (2000). Mid- and Near-Infrared Investigation of Molecular Orientation in Elastomeric Networks. Appl. Spectrosc..

[cit52] Ye H., Gu Z., Gracias D. H. (2006). Kinetics of Ultraviolet and Plasma Surface Modification of Poly(dimethylsiloxane) Probed by Sum Frequency Vibrational Spectroscopy. Langmuir.

[cit53] Egitto F. D., Matienzo L. J. (2006). Transformation of Poly(dimethylsiloxane) into thin surface films of SiO_*x*_ by UV/Ozone treatment. Part I: Factors affecting modification. J. Mater. Sci..

[cit54] Gunde M. K. (2000). Vibrational modes in amorphous silicon dioxide. Phys. B.

[cit55] Crampton A. S., Ridge C. J., Rötzer M. D. (2015). *et al.*, Atomic Structure Control of Silica Thin Films on Pt(111). J. Phys. Chem. C.

[cit56] Brongersma H., Draxler M., Deridder M., Bauer P. (2007). Surface composition analysis by low-energy ion scattering. Surf. Sci. Rep..

[cit57] Cushman C. V., Brüner P., Zakel J. (2016). *et al.*, Low energy ion scattering (LEIS). A practical introduction to its theory, instrumentation, and applications. Anal. Methods.

[cit58] Brongersma H. H., Grehl T., Schofield E. R., Smith R. A. P., Ter Veen H. R. J. (2010). Analysis of the Outer Surface of Platinum-Gold Catalysts by Low-Energy Ion Scattering:Improved resolution allows selective analysis of mixed metal systems. Platinum Met. Rev..

[cit59] Pra S., Linford M. R., Vaníková E. (2024). *et al.*, A practical guide to interpreting low energy ion scattering (LEIS) spectra. Appl. Surf. Sci..

[cit60] Innocenzi P. (2003). Infrared spectroscopy of sol–gel derived silica-based films: a spectramicrostructure overview. J. Non-Cryst. Solids.

[cit61] Mazarei A. F., Sandall O. C. (1980). Diffusion coefficients for helium, hydrogen, and carbon dioxide in water at 25 °C. AIChE J..

[cit62] Levich V. G. , Physicochemical Hydrodynamics, Prentice-Hall International Series in the Physical and Chemical Engineering Sciences, Prentice-Hall, Englewood Cliffs, NJ, 1962, p. 700

[cit63] Ultrathin Oxide Layers for Solar and Electrocatalytic Systems, ed. H. Frei and D. V. Esposito, Royal Society of Chemistry, 2022, ISBN: 9781839161797

[cit64] Wei C., Rao R. R., Peng J. (2019). *et al.*, Recommended Practices and Benchmark Activity for Hydrogen and Oxygen Electrocatalysis in Water Splitting and Fuel Cells. Adv. Mater..

[cit65] Johnson L., Ejigu A., Licence P., Walsh D. A. (2012). Hydrogen Oxidation and Oxygen Reduction at Platinum in Protic Ionic Liquids. J. Phys. Chem. C.

[cit66] Beatty M. E. S., Gillette E. I., Haley A. T., Esposito D. V. (2020). Controlling the Relative Fluxes of Protons and Oxygen to Electrocatalytic Buried Interfaces with Tunable Silicon Oxide Overlayers. ACS Appl. Energy Mater..

[cit67] Bau J. A., Takanabe K. (2017). Ultrathin Microporous SiO= Membranes Photodeposited on Hydrogen Evolving Catalysts Enabling Overall Water Splitting. ACS Catal..

[cit68] Jin J., Cohen L. A., Adibnia S. (2026). *et al.*, Proton Conducting Silicon Oxide Membranes as a Fluorine Free Alternative to Nafion for Low Temperature Water Electrolysis. ACS Appl. Mater. Interfaces.

[cit69] Ahmadpour S., Gholami R., Ghaedi M., Blunt M. J. (2025). An experimental study and mathematical formulation for hydrogen diffusion in water. Sci. Rep..

[cit70] López-Cudero A., Cuesta A., Gutiérrez C. (2005). Potential dependence of the saturation CO coverage of Pt electrodes: The origin of the pre-peak in CO-stripping voltammograms. Part 1: Pt(111). J. Electroanal. Chem..

[cit71] Wang R. , Low Carbon Steam Reforming-Based Hydrogen Production, Tech. rep. Gas Liquids Engineering Ltd, 2020

[cit72] U.S. Department of Energy, Hydrogen, Fuel Cells, and Infrastructure Technologies: FY 2002 Progress Report, Tech. rep. Office of Energy Efficiency and Renewable Energy, 2002

[cit73] U.S. Department of Energy, Fuel Cells for Transportation, Tech. rep. Alternative Fuels Data Center, 1999

[cit74] Pedroso O. , Champion Y., Botta W. and Zepon G., Johnson-Mehl-Avrami-Kolmogorov model applied to describe the site blocking effect in interstitial solid solution, *ChemRxiv*, 2023, preprint 10.26434/chemrxiv-2023-8d22n

[cit75] Sayed-Ahmed H., Toldy Á. I., Santasalo-Aarnio A. (2024). Dynamic operation of proton exchange membrane electrolyzers—Critical review. Renewable Sustainable Energy Rev..

