# Supplementary material for: CO-tolerant hydrogen oxidation on Pt enabled by ultrathin silica overlayers
Source: EES Catal. 2026 Aug 24;4(6):1336–50. doi: 10.1039/d6ey00101g (PMC13573285; doi:10.1039/d6ey00101g)
Supplement: Supplementary file 2 [file EY-004-D6EY00101G-s001.pdf]

Supplementary Information (SI) for EES Catalysis.
This journal is © The Royal Society of Chemistry 2026

Supporting Information for: CO-Tolerant Hydrogen Oxidation on Pt Enabled by Ultrathin Silica Overlayers

Christos Englezos[1], Marco Altomare[1], Guido Mul[1], Bastian Mei*[1,2], and Georgios Katsoukis*[1]

[1]MESA+ Institute of Nanotechnology, Department of Chemical Engineering, Faculty of Science and Technology, University of Twente, Drienerlolaan 5, 7522 NB Enschede, The Netherlands
[2]Fakultät für Chemie & Biochemie Technische Chemie Energie und Umweltrelevante Katalyse, Ruhr-Universität Bochum, Germany

Corresponding authors:
Georgios Katsoukis[1]; Bastian Mei[1,2]

Email addresses: g.katsoukis@utwente.nl; bastian.mei@rub.de

Contents

1 3D areal surface topography (confocal microscopy) of the Ti substrate 2

2 Pt ECSA cyclic voltammograms (CVs) of 50nm-Pt|Ti, edep-Pt|Ti electrodes and SiO_x-coated edep-Pt|Ti electrodes 3

3 XRR-IRRAS cross-calibration for silica thickness determination 5

4 Extraction of D_{H_2} in solution and H_2 permeability through SiO_x from rotation-dependent limiting currents 8

5 Effect of prolonged UV–Ozone treatment on PDMS-to-SiO_x conversion 13

6 Replicate chronoamperometry (CA) traces 14

7 Determination of the Pt ECSA by CO-Stripping Voltammetry 15

8 LEIS spectra of the silica-coated edep-Pt|Ti surface before and after long CO exposure 17

9 Non-linear CO poisoning model fitting and analysis 18

10 CO-poisoning CA of atomic layer deposition (ALD) SiO_2 on edep-Pt|Ti 22

1 3D areal surface topography (confocal microscopy) of the Ti substrate

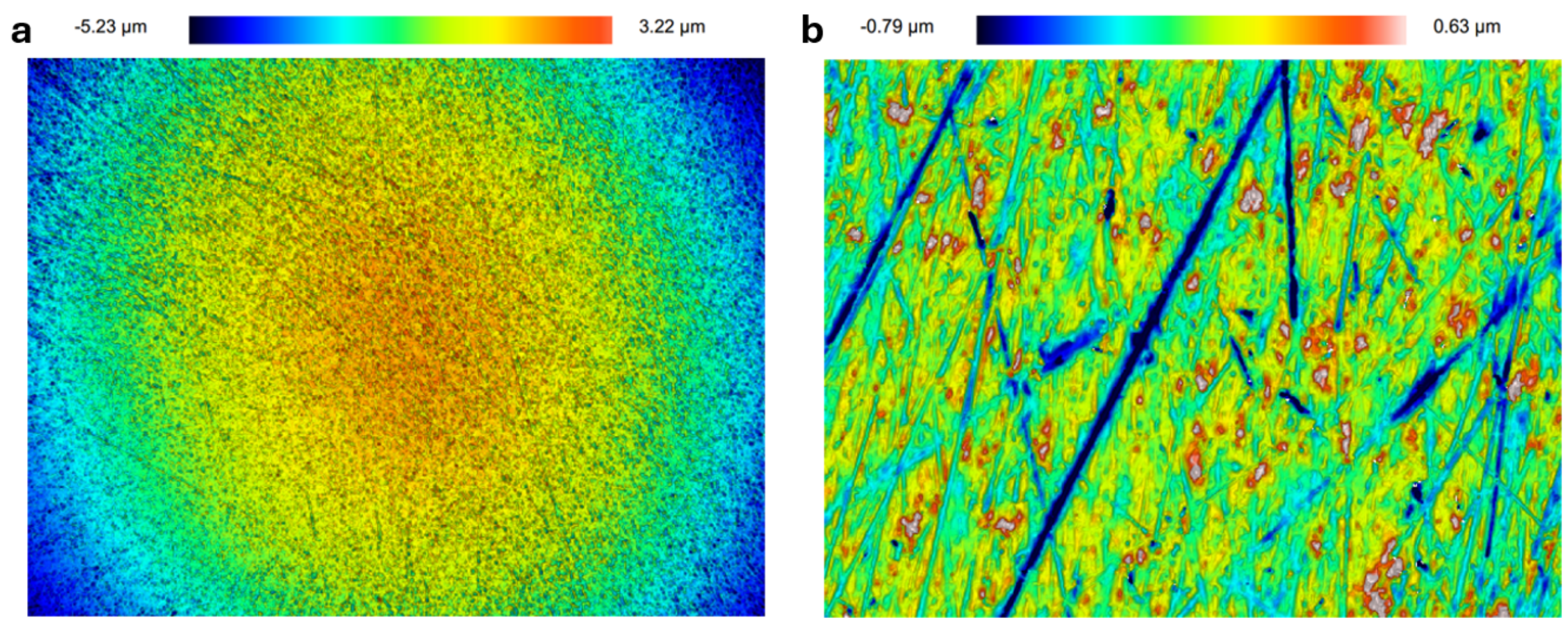

Figure S1. Top-view height maps acquired with a Sensofar S neox confocal microscope and evaluated according to ISO 25178 after plane form removal. **(a)** Polished Ti disk, wide-area 10× scan (2.3 mm^2 probed): average areal roughness $S_a \approx 1.07$ µm, total height $S_z \approx 8.4$ µm, and negative skewness $S_{sk} \approx -0.43$, consistent with a plateau-like morphology dominated by deeper valleys relative to peaks. **(b)** Polished Ti disk, high-magnification 100× scans (three random locations; 0.23 mm^2 total probed): fine-scale roughness $S_a = (68 \pm 7)$ nm, indicating a relatively smooth micro-plateau with isolated micro-scale asperities. Color bars indicate height in µm (note that each panel uses its own height range).

2 Pt ECSA cyclic voltammograms (CVs) of 50nm-Pt|Ti, edep-Pt|Ti electrodes and SiO_x-coated edep-Pt|Ti electrodes

Electrochemically active surface area (ECSA) values were estimated from the CVs in Figure S2 using the charge associated with hydrogen underpotential deposition (H_{upd}) desorption. Specifically, the anodic H_{upd} desorption region was integrated from 0.03 to 0.45 V vs. RHE after subtraction of the double-layer background current-estimated graphically, and the resulting charge was converted to ECSA using the commonly accepted value of 210 μC cm_{Pt}^{-2} for polycrystalline Pt:

$$\mathrm{ECSA} = \frac{Q_{\mathrm{H_{upd}},\mathrm{des}}}{210\ \mu\mathrm{C\ cm_{Pt}^{-2}}} \tag{S1}$$

where $Q_{\mathrm{H_{upd}},\mathrm{des}}$ is the double-layer background current-corrected H_{upd} desorption charge.

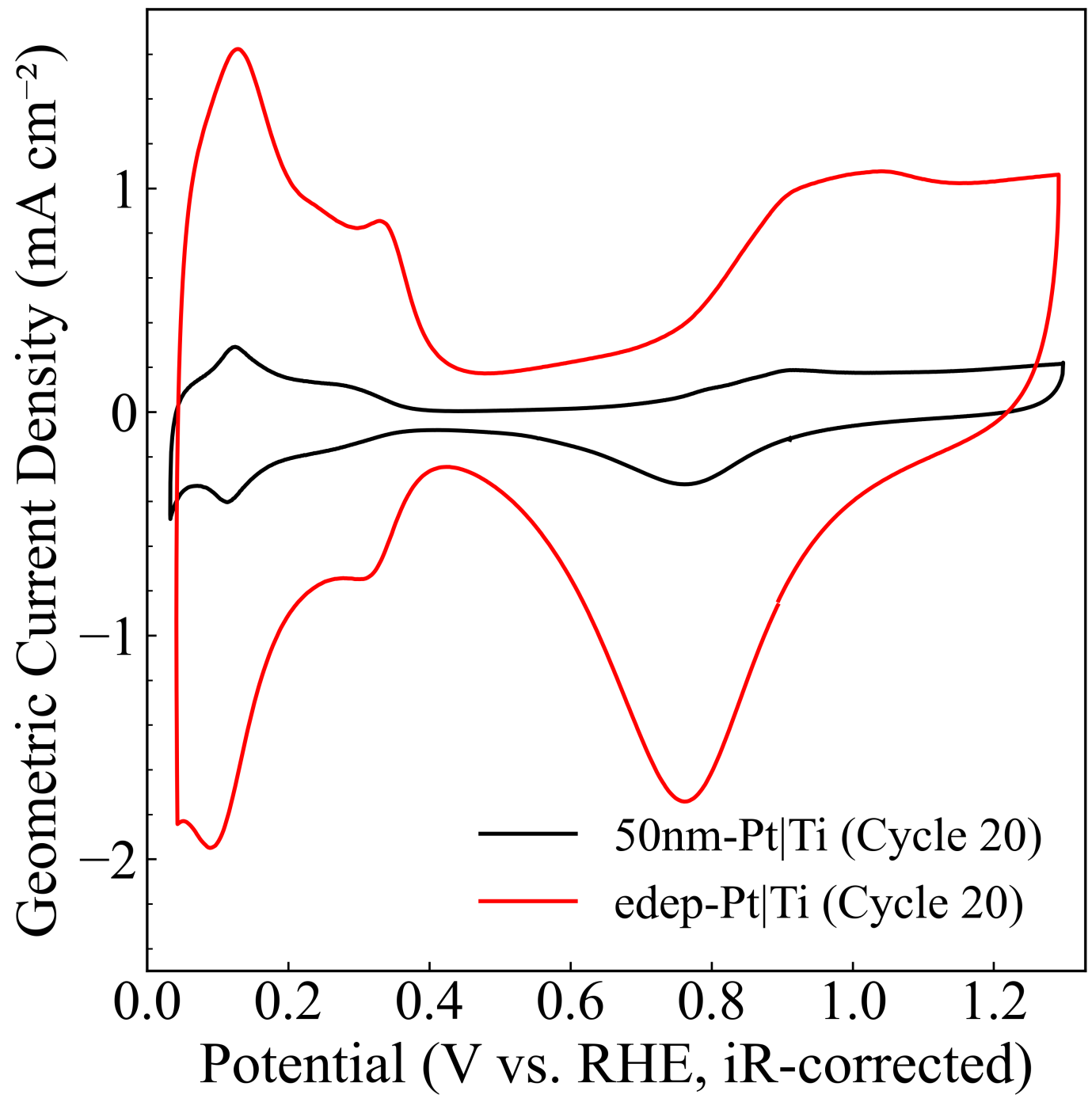

Figure S2. Cyclic voltammograms of 50nm-Pt|Ti (black) and edep-Pt|Ti (red), shown as geometric current density versus iR-corrected potential (V vs. RHE). The 20th cycle is shown for each sample, recorded at 100 mV s^{-1}. Electrolyte for all measurements: 0.1 M $HClO_4$ (pH = 1.1) at ambient conditions.

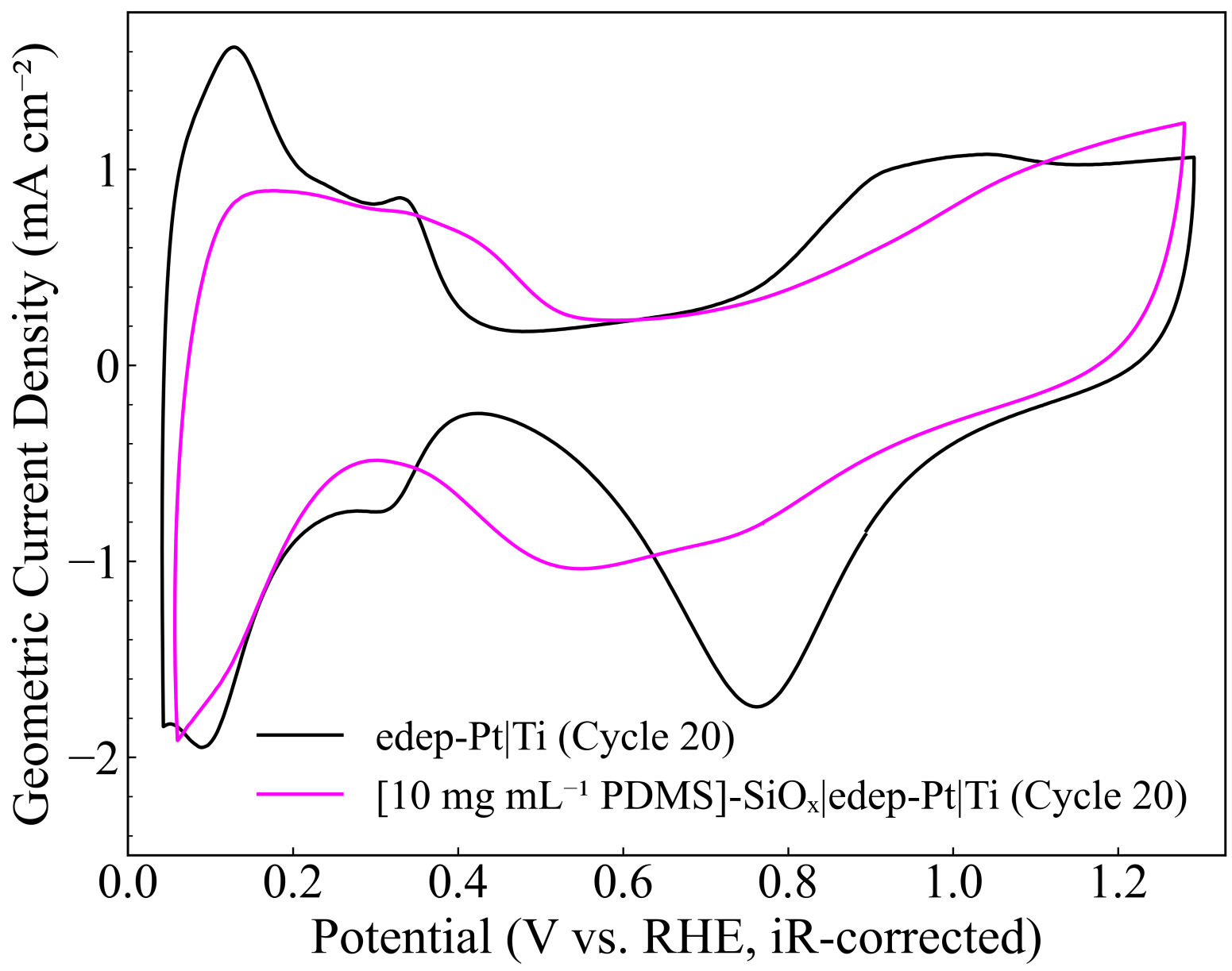

Figure S3. Cyclic voltammograms of uncoated edep-Pt|Ti and SiO_x-coated edep-Pt|Ti electrodes, shown as geometric current density versus iR-corrected potential (V vs. RHE). The 20th cycle is shown for each sample, recorded at 100 mV s^{-1}. Electrolyte for all measurements: 0.1 M $HClO_4$ (pH = 1.1) at ambient conditions.

3 XRR-IRRAS cross-calibration for silica thickness determination

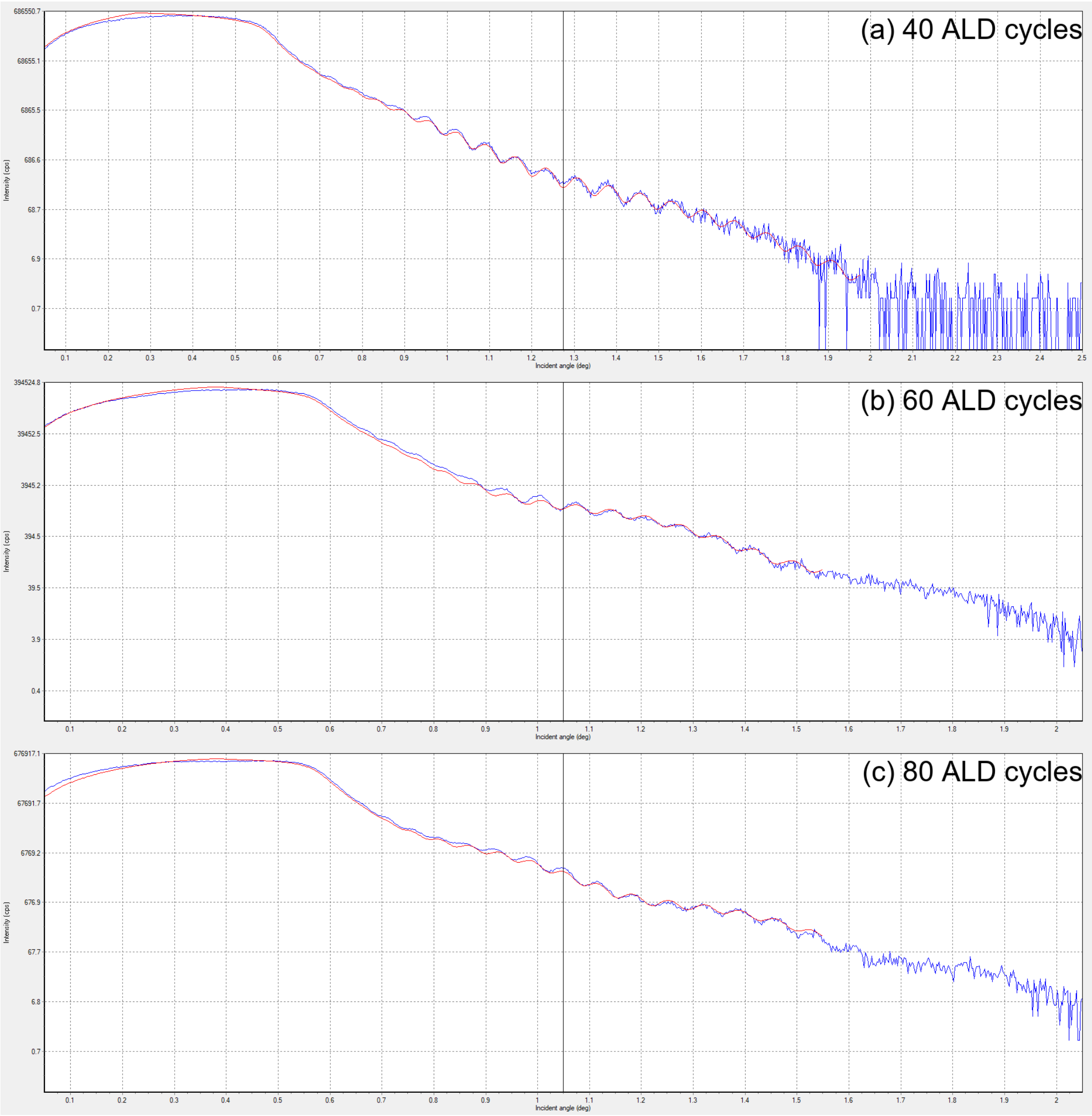

Figure S4. X-ray reflectivity (XRR) experimental data and corresponding fitted models for SiO_2 films grown by atomic layer deposition using bis(diethylamino)silane (BDEAS) and ozone at 200 °C on substrates consisting of 50 nm sputtered Pt on 100 nm thermal SiO_2/Si wafers, for (a) 40, (b) 60, and (c) 80 ALD cycles. The fits were used to extract the film thickness values reported in Figure S5. Measurements were performed using a Cu Kα radiation source ($\lambda = 1.5406$ Å), and the experimental data were fitted using a multilayer slab model.

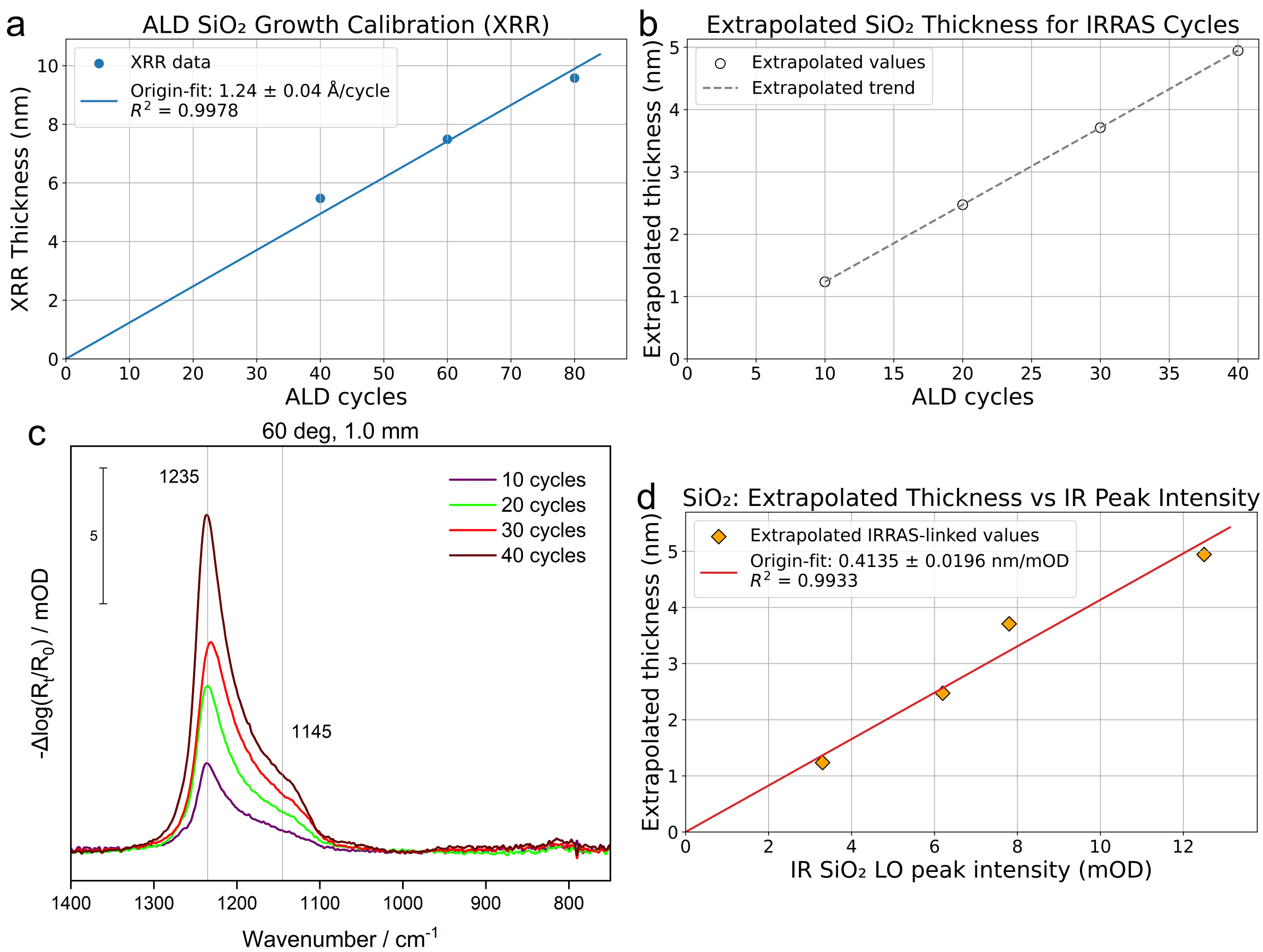

Figure S5. SiO_2 thickness calibration by XRR and IRRAS. **(a)** XRR-derived SiO_2 thickness as a function of ALD cycle number. Filled circles denote measured XRR thicknesses at 40, 60, and 80 cycles; XRR fitting is reliable for films thicker than approximately 5 nm. A linear regression constrained to pass through the origin yields a growth-per-cycle (GPC) of 1.24 ± 0.04 Å/cycle with $R^2 = 0.9978$. **(b)** SiO_2 thicknesses extrapolated from the XRR calibration in panel (a) for lower ALD cycle numbers relevant to IRRAS measurements (10, 20, 30, and 40 cycles). Open circles denote the extrapolated thickness values, and the dashed line indicates the extrapolated trend. **(c)** IRRAS spectra tracking the evolution of the surface species with increasing ALD cycle number (10, 20, 30, and 40 cycles), corresponding to films with thicknesses below 5 nm. Spectra are shown as background-subtracted reflectance absorbance, $-\Delta \log(R_t/R_0)$, in milli-optical density units (mOD) versus wavenumber. Measurements were collected using a 1 mm aperture at a grazing angle of 60°. The prominent bands centered near 1235 (LO mode) and 1145 (TO mode) cm^{-1} increase with cycle number and are consistent with growth of the silica-based film. **(d)** Correlation between extrapolated SiO_2 thickness from panel (b) and the IR SiO_2 LO peak intensity. Diamond symbols denote the extrapolated IRRAS-linked values. A linear regression constrained through the origin gives thickness $= (0.4135 \pm 0.0196)$ nm/mOD $\times$ intensity, with $R^2 = 0.9933$, enabling conversion of IR peak intensity to film thickness.

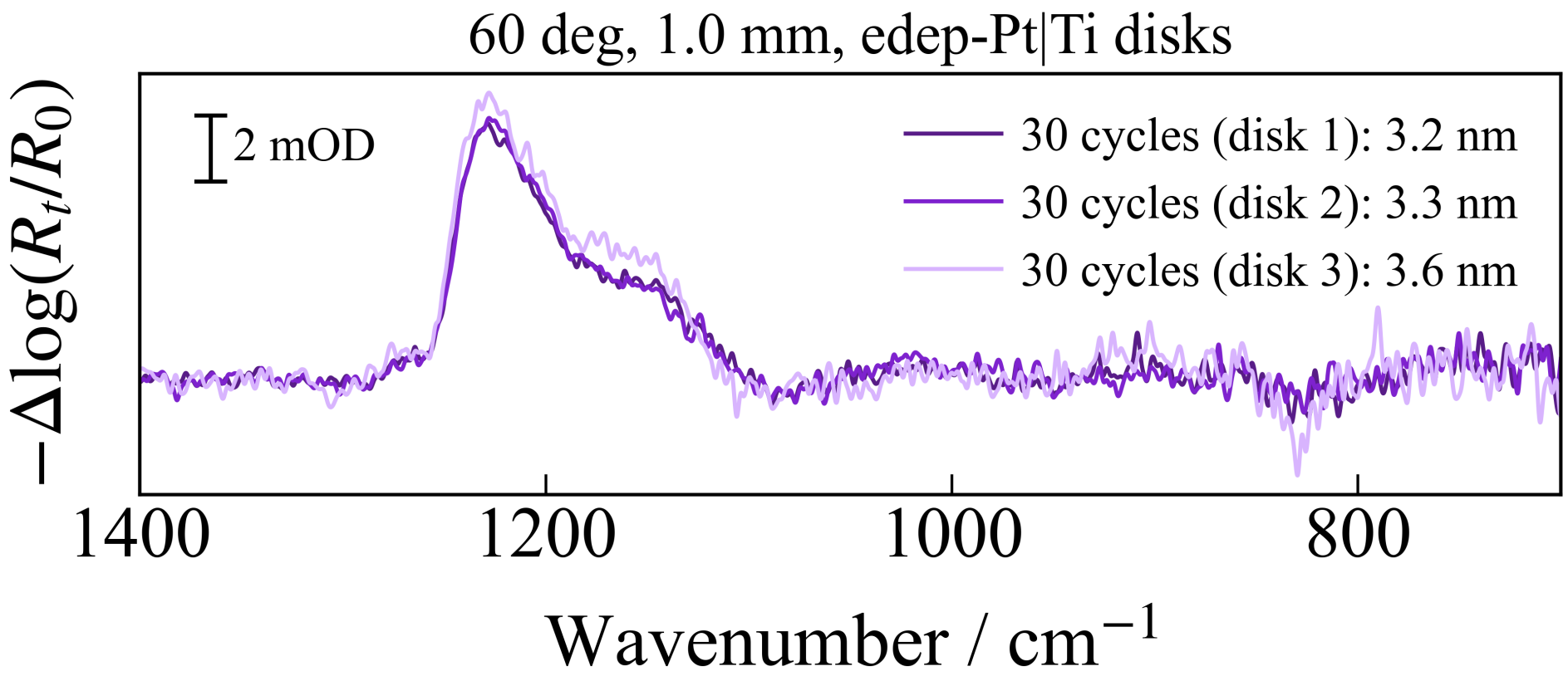

Figure S6. IRRAS spectra of three independently prepared edep-Pt|Ti disks coated with 30 ALD cycles of SiO_2. The wafer-derived calibration gives an average thickness of 3.4 ± 0.2 nm, in good agreement with the nominal ALD thickness of approximately 3.6 nm.

4 Extraction of D_{H_2} in solution and H_2 permeability through SiO_x from rotation-dependent limiting currents

In the present electrochemical system, mass-transfer resistances arise both in the solution-side diffusion boundary layer (DBL) and in the thin overlayer that coats the Pt electrode. Under steady state, the molar flux of any species is conserved, hence the flux through the DBL equals that through the overlayer, i.e. the same current density must be maintained at both regions.

Considering the oxidant O as the transport-limiting species, the current density i can be written with finite-difference forms of Ficks law for each region. Across the DBL of thickness δ and diffusivity D_{H_2}, the current is driven by the drop from the bulk concentration $C_{H_2,b}$ to the interfacial concentration at the overlayer/electrolyte boundary, $C_{H_2}(x = l_{ov})$. Across the overlayer of thickness l_{ov}, transport is characterized by the permeability P_{H_2} of H_2 (the product of an effective diffusivity and a partition coefficient). Taking the conventional sign for oxidation currents (HOR), the steady-state equal i for each region are:

$$i = nF\,\frac{D_{H_2}}{\delta}\left[C_{H_2,b} - C_{H_2}(x = l_{ov})\right] = nF\,\frac{P_{H_2}}{\delta}\left[C_{H_2}(x = l_{ov}) - C_{H_2}(x = 0)\right] \tag{S2}$$

where the second expression assumes the concentration at the inner overlayer/electrode interface is negligible relative to $C_{H_2}(x = l_{ov})$, under transport-limited conditions.[1] Eliminating $C_{H_2}(x = l_{ov})$ yields a closed form for the limiting current:

$$i_{\mathrm{lim}} = -nF\,C_{H_2,b}\left(\frac{\delta}{D_{H_2}} + \frac{l_{ov}}{P_{H_2}}\right)^{-1} \tag{S3}$$

Defining the isolated limiting currents due solely to the DBL and solely to the overlayer as follows:

$$i_{\mathrm{lim,DBL}} = nF\,\frac{D_{H_2}}{\delta}\,C_{H_2,b} \tag{S4}$$

$$i_{\mathrm{lim},ov} = nF\,\frac{P_{H_2}}{l_{ov}}\,C_{H_2,b} \tag{S5}$$

the overall limiting current satisfies a resistances-in-series structure, which is basically the reciprocal rearrangement of Equation S3:

$$\frac{1}{i_{\mathrm{lim}}} = \frac{1}{i_{\mathrm{lim,DBL}}} + \frac{1}{i_{\mathrm{lim},ov}} \tag{S6}$$

Since rotational experiments are performed (RDE), the DBL of thickness δ and diffusivity D_{H_2} is well defined and the limiting current density for this region can be described by the Levich equation:

$$i_{lim,DBL} \;=\; 0.201\, n F A D^{2/3} \nu^{-1/6} C^{*} \omega^{1/2} = B\,\omega^{1/2} \tag{S7}$$

with n the stoichiometric number of electrons transferred per molecule (dimensionless, 2 for HOR), F the Faraday constant (9.6485×10^4 C mol^{-1}), A the geometric area of the disk electrode (0.196 cm^2), D_{H_2} the diffusion coefficient of H_2 in the solution (cm^2 s^{-1}), ν the kinematic viscosity of water at 23žC (9.33×10^{-3} cm^2 s^{-1}), C^* the bulk concentration of H_2 in water at 23žC (7.9×10^{-7} mol cm^{-3}), ω the angular rotation rate of the disk in rpm. Performing LSV experiments, the current is recorded for a fixed potential range at a specific rotation rate, each LSV curve corresponds to a specific rotation. In the Levich equation, the only variable then is the angular rotation rate of the disk in rpm and the rest of the parameters are constants that can be condensed to a single constant B, the Levich constant. Substituting eq.S7 and eq.S4 in eq.S6, we derive[1]:

$$\frac{1}{i_{\mathrm{lim}}} = \left(\frac{1}{i_{\mathrm{lim},ov}}\right) + \left(\frac{1}{B}\right)\frac{1}{\omega^{1/2}} \tag{S8}$$

Plotting the experimental current vs. rotation rate (LSV curves) in their reciprocal values, linear fitting gives the slope $1/B$ and the intercept $1/_{lim,ov}$. From the slope estimate the diffusion coefficient of H_2, D_{H_2} in solution is calculated. Then, from the intercept estimate, the permeability of H_2 through the overlayer can be calculated provided that the overlayer thickness is known[2,3].

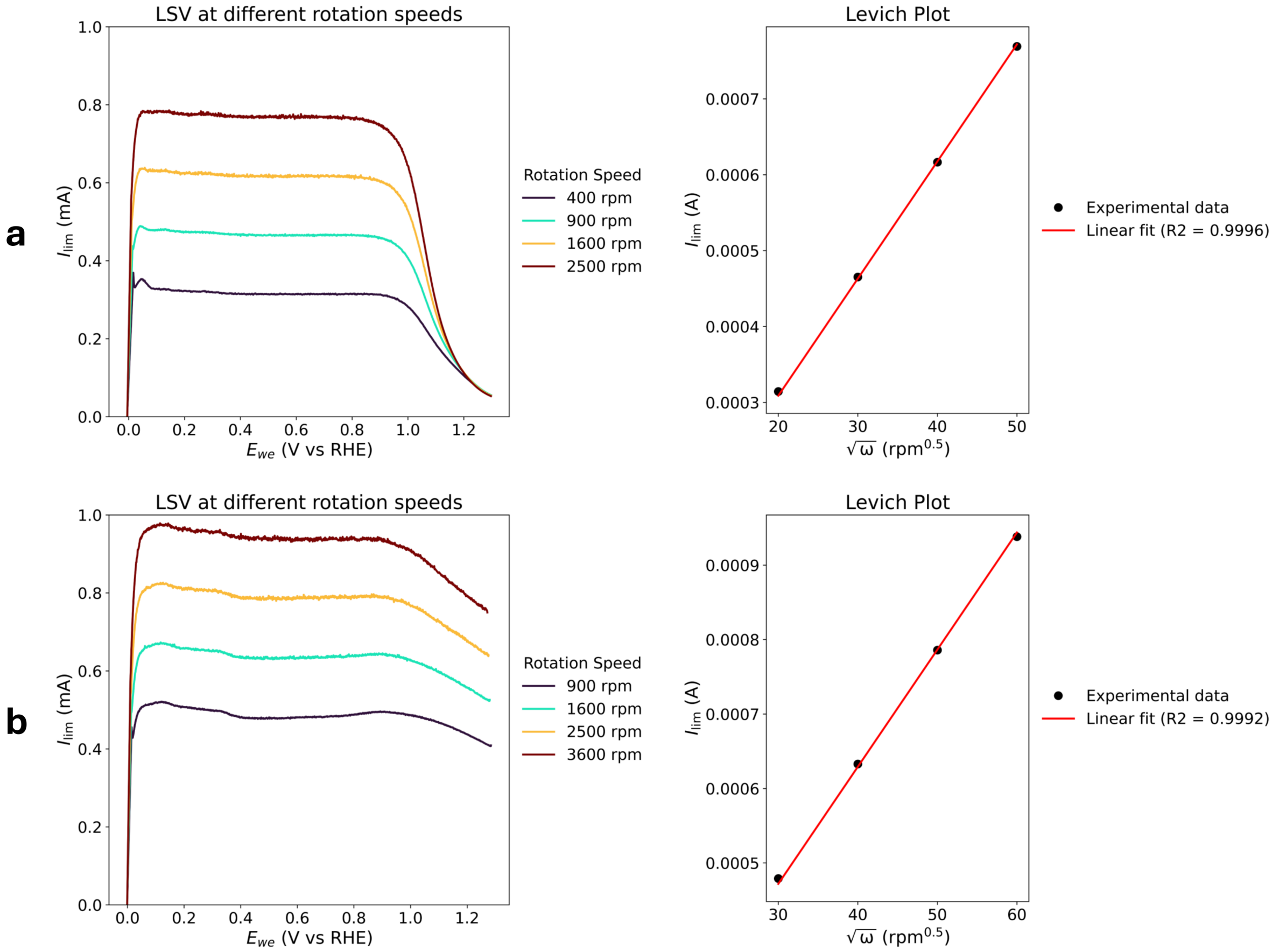

Figure S7. Linear sweep voltammograms (LSVs) recorded at varying rotation rates in H_2-saturated 0.1 M $HClO_4$ and corresponding linear regression of the experimental data in i_{lim} vs. $\omega^{1/2}$ (Levich plot), for uncoated 50nm-Pt|Ti **(a)**, and uncoated edep-Pt|Ti **(b)**. Currents were corrected for ohmic drop prior to analysis. For each rotation rate, the *average limiting current* i_{lim} was extracted from the fully developed limiting-current plateau between 0.5–0.6 V vs. RHE, where the response is governed by H_2 transport. The extracted parameters from the Levich slope (no intercept) is diffusivity D_{H_2} in the electrolyte.

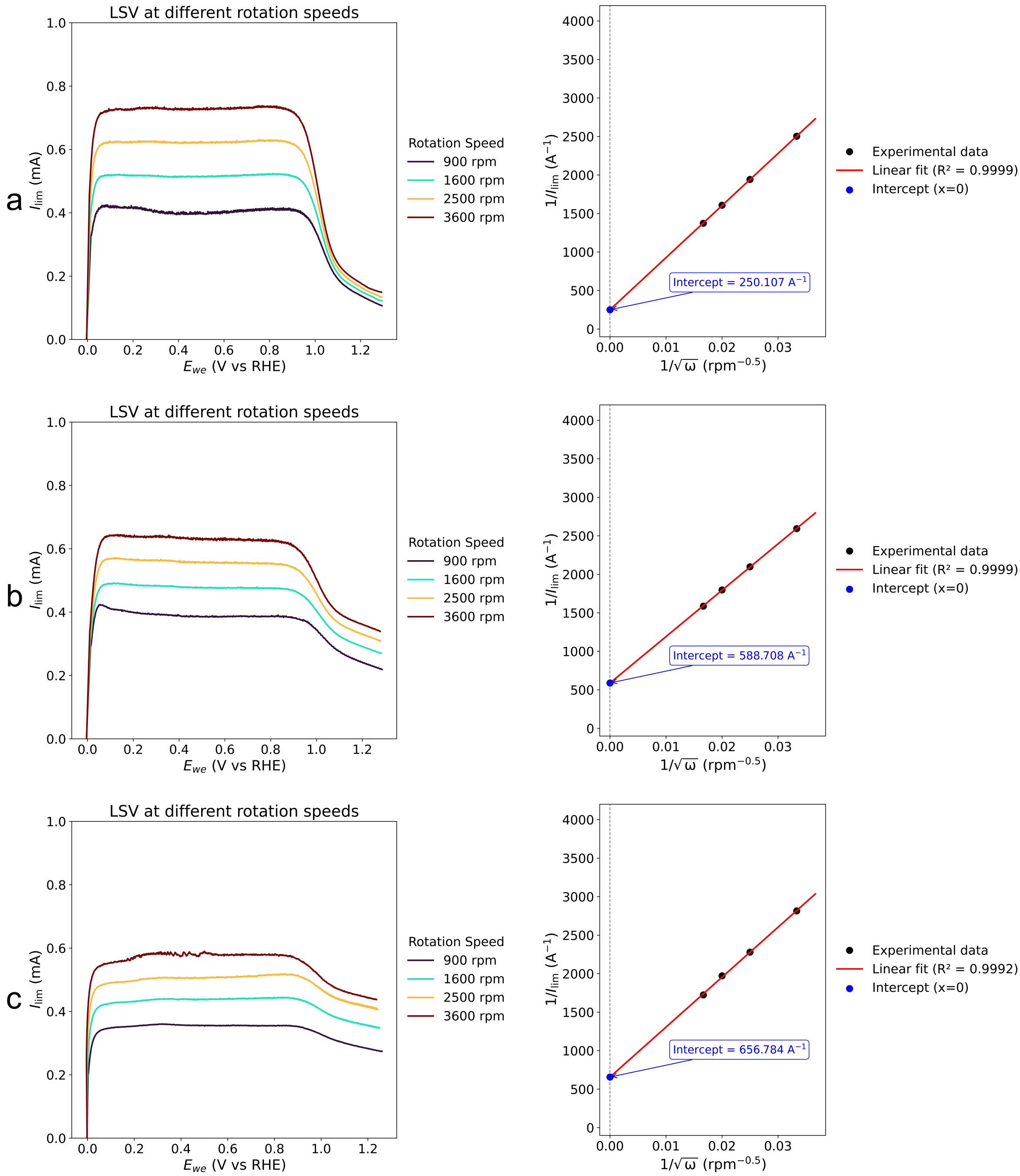

Figure S8. Linear sweep voltammograms (LSVs) recorded at varying rotation rates in H_2-saturated 0.1 M $HClO_4$ and corresponding linear regression of the experimental data in $1/i_{\text{lim}}$ vs. $1/\omega^{1/2}$, for SiO_x-coated 50nm-Pt|Ti using 5 **(a)**, 10 **(b)**, and 20 **(c)** mg mL^{-1} PDMS. Currents were corrected for ohmic drop prior to analysis. For each rotation rate, the *average limiting current* i_{lim} was extracted from the fully developed limiting-current plateau between 0.5–0.6 V vs. RHE, where the response is governed by H_2 transport. The extracted parameters from the slope and intercept are diffusivity D_{H_2} in the electrolyte and effective permeability P_{H_2} through the SiO_x overlayer respectively.

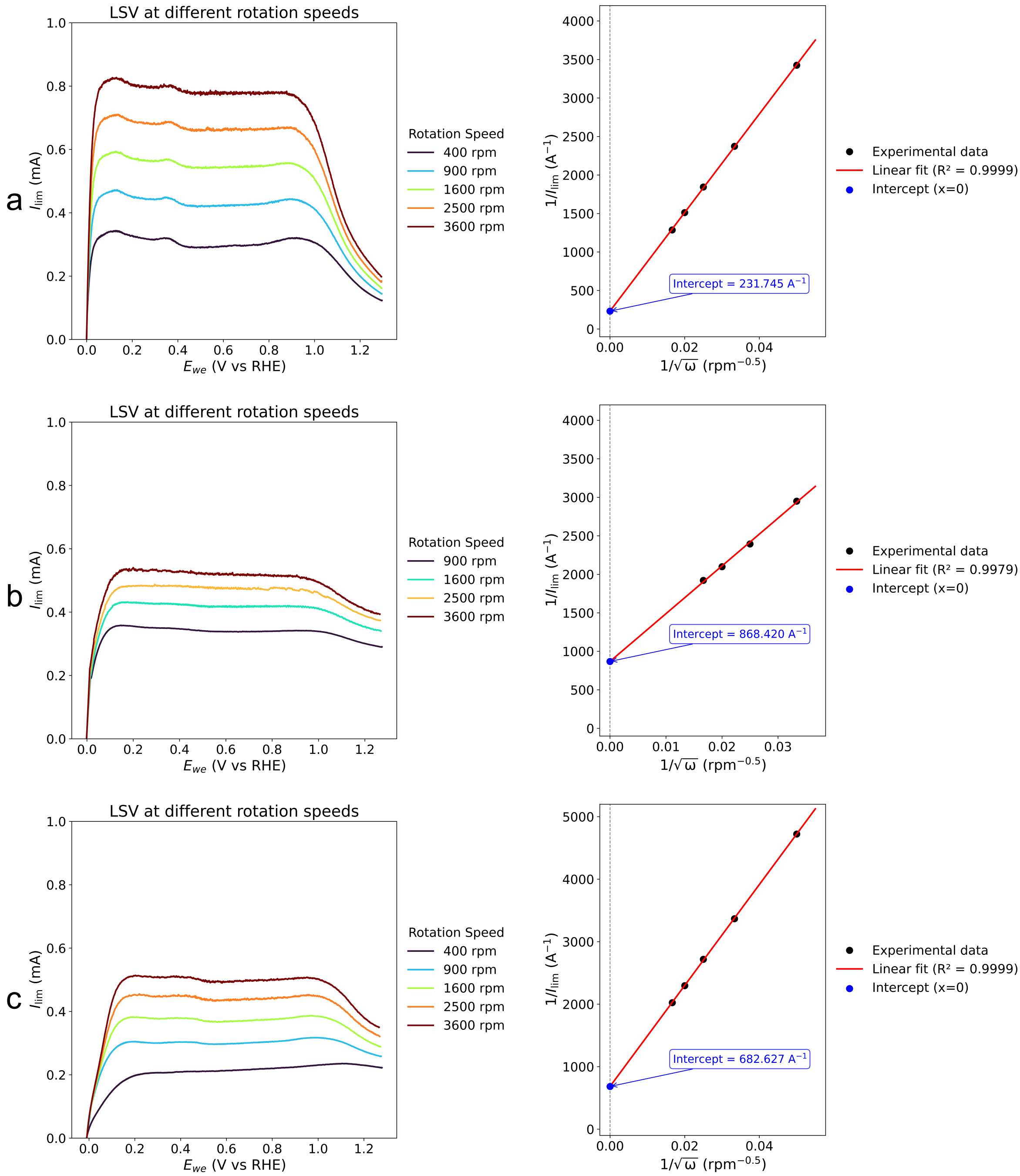

Figure S9. Linear sweep voltammograms (LSVs) recorded at varying rotation rates in H_2-saturated 0.1 M $HClO_4$ and corresponding linear regression of the experimental data in $1/i_{\text{lim}}$ vs. $1/\omega^{1/2}$, for SiO_x-coated edep-Pt|Ti using 5 **(a)**, 10 **(b)**, and 20 **(c)** mg mL^{-1} PDMS. Currents were corrected for ohmic drop prior to analysis. For each rotation rate, the *average limiting current* i_{lim} was extracted from the fully developed limiting-current plateau between 0.5–0.6 V vs. RHE, where the response is governed by H_2 transport. The extracted parameters from the slope and intercept are diffusivity D_{H_2} in the electrolyte and effective permeability P_{H_2} through the SiO_x overlayer respectively.

5 Effect of prolonged UV–Ozone treatment on PDMS-to-SiO_x conversion

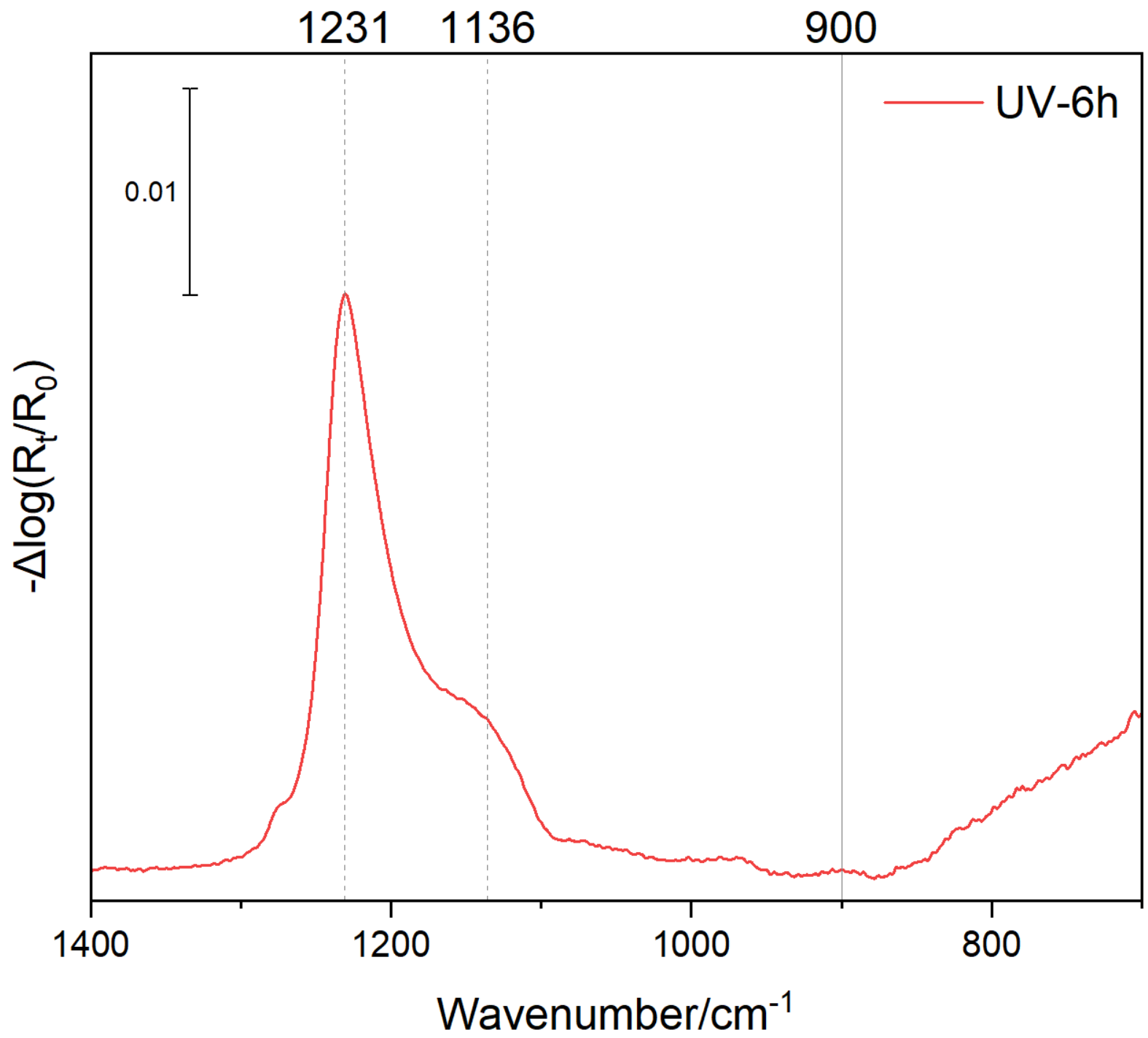

Figure S10. Infrared reflection–absorption spectrum (IRRAS) of a 10 mg mL^{-1} PDMS-derived SiO_x thin film on edep-Pt|Ti after prolonged UV–ozone treatment for 6 h, showing persistence of a minor residual methyl-related feature. The measurement was collected using a 1.0 mm aperture at an incidence angle of 60°. mOD: milli-optical density units.

6 Replicate chronoamperometry (CA) traces

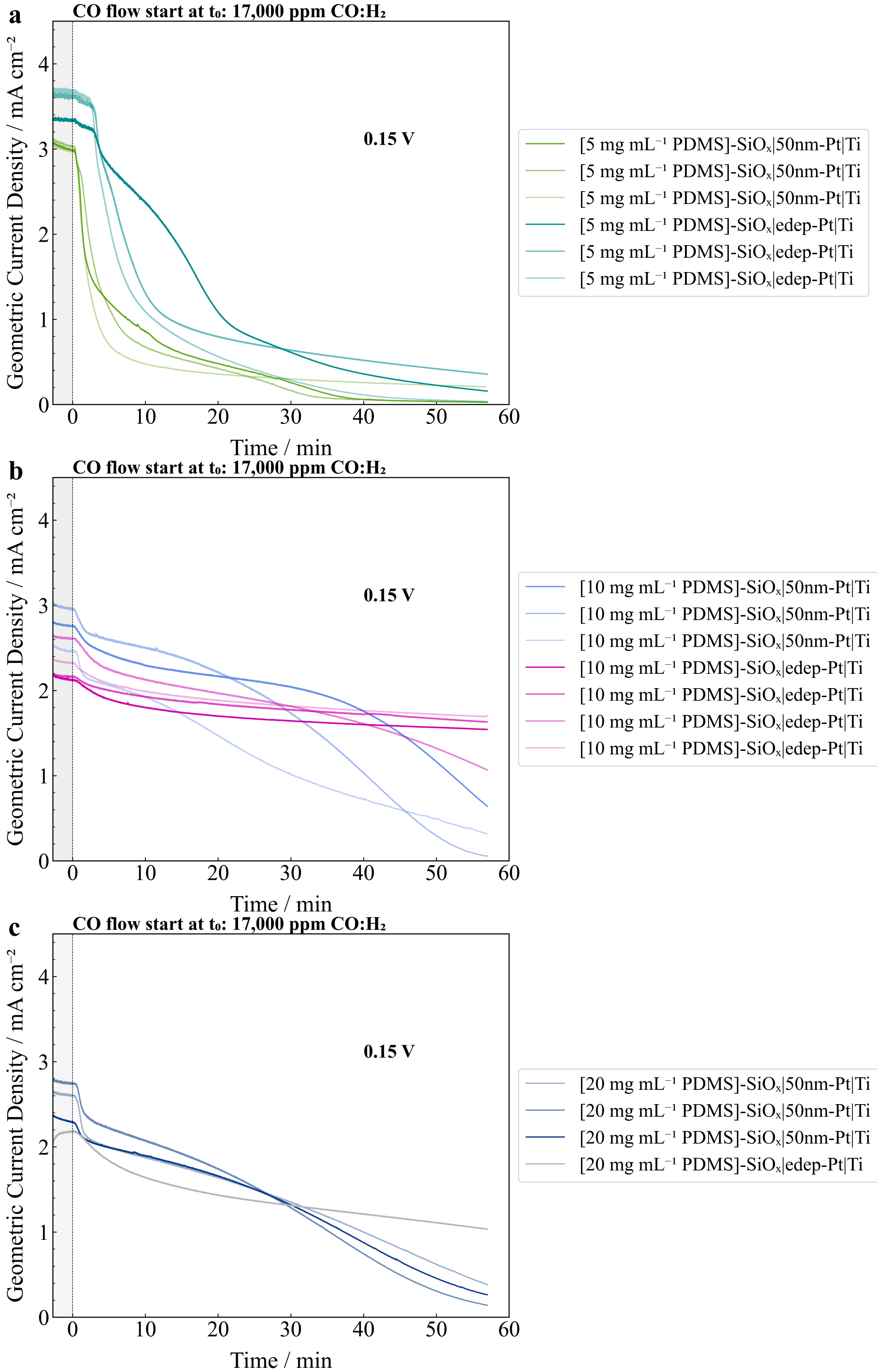

Figure S11. Complete CA trace dataset for all SiO$_x$ coated electrodes prepared with different PDMS precursor concentrations:(a) 5, (b) 10, (c) 20 mg mL^{-1} PDMS for 50nm- and edep-Pt.

7 Determination of the Pt ECSA by CO-Stripping Voltammetry

The electrochemically active Pt surface area was determined by CO-stripping voltammetry. The CO-stripping scans were initiated immediately after the CO-poisoning chronoamperometry. At the end of the CA measurement, the H_2/CO gas flow was stopped and the voltammetric scan was started without an intermediate inert-gas purge. Consequently, dissolved H_2 remained in the electrolyte during the initial stripping cycles, giving rise to an additional HOR in the case of the silica coated electrode being not fully poisoned (after 1h of CA), and Pt sites remaining accessible after CO exposure. This accounts for the additional anodic current between approximately 0.1 and 0.4 V vs. RHE. The anodic branch of Cycle 2, recorded after removal of the majority of adsorbed CO during Cycle 1, was therefore first interpolated onto Cycle 1 and subtracted to remove the electrochemical background. Because the dissolved H_2 concentration and Pt surface state change between the two cycles, a slowly varying residual background remained after this subtraction. For determination of the CO-related oxidation charge, a local linear baseline was constructed between the mean residual currents at the lower and upper limits of the integration interval (0.50-0.90 V vs. RHE, 10 points at each boundary) and subtracted from the Cycle 1–Cycle 2 difference. Only the positive background-residual baseline-corrected current within this interval was integrated and converted to the total CO-stripping charge, Q_{CO}, using the applied scan rate. The electrochemically active Pt area was calculated according to the following relation where a charge density of 420 $\mu\mathrm{C\,cm_{Pt}^{-2}}$ was assumed for the oxidation of a complete CO monolayer on Pt:

$$A_{\mathrm{ECSA,CO}} = \frac{Q_{\mathrm{CO}}}{420\ \mu\mathrm{C\,cm_{Pt}^{-2}}}, \tag{S9}$$

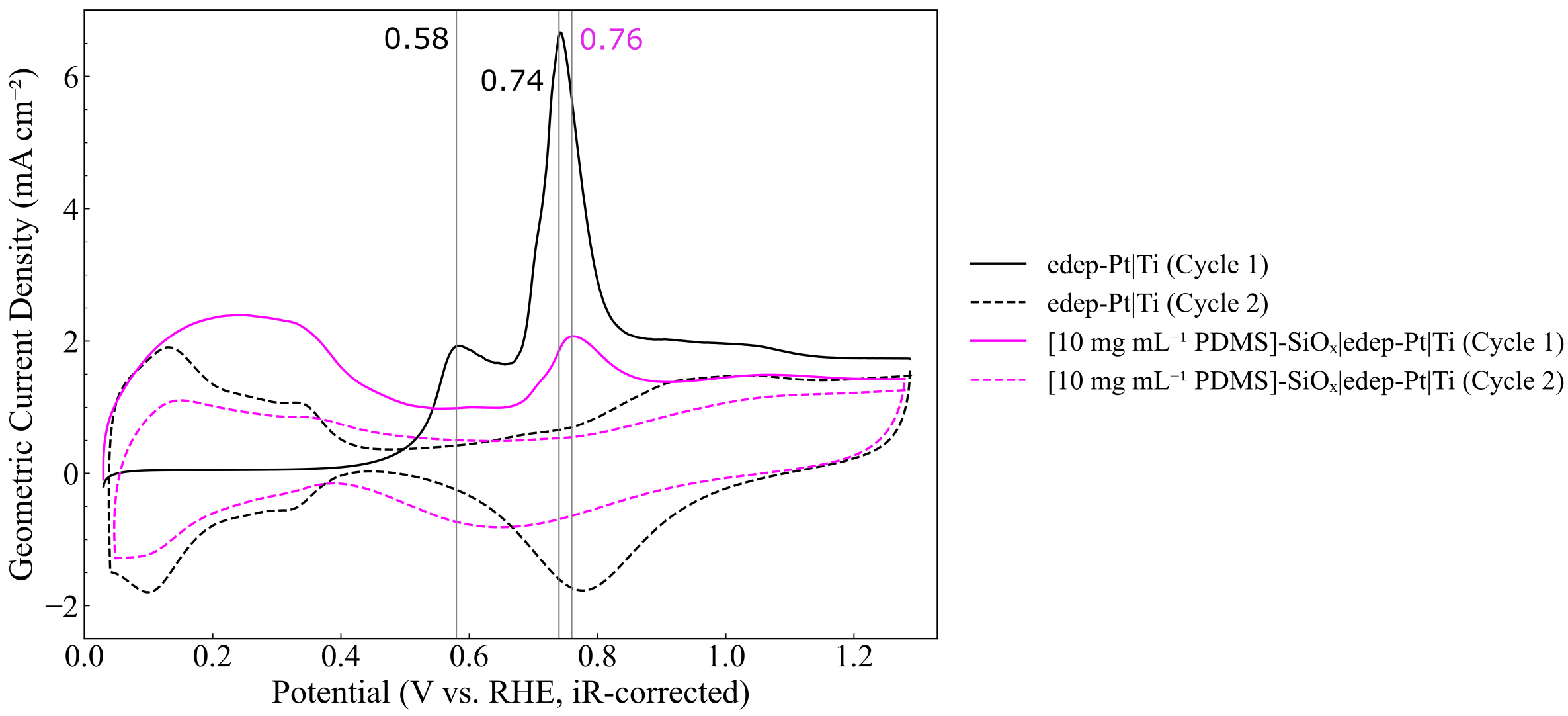

Figure S12. CO-stripping voltammograms of uncoated edep-Pt|Ti and SiO_x-coated edep-Pt|Ti electrodes, shown as geometric current density versus iR-corrected potential (V vs. RHE). The first and second cycles are shown as solid and dashed lines, respectively. Electrolyte for all measurements: 0.1 M $HClO_4$ (pH = 1.1) at ambient conditions.

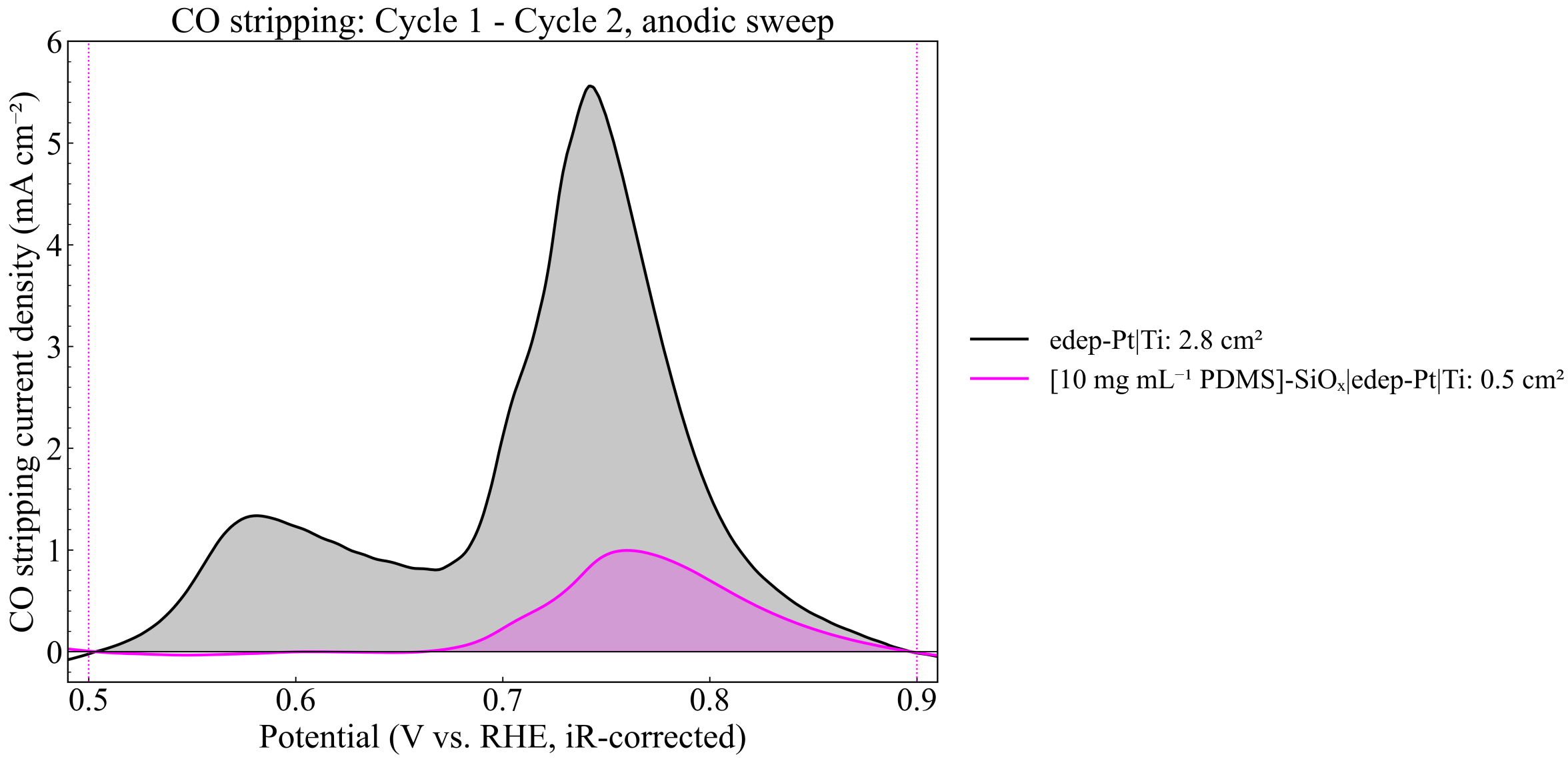

Figure S13. Background-residual baseline-corrected CO-stripping profiles of uncoated edep-Pt|Ti and SiO_x-coated edep-Pt|Ti electrodes, obtained by subtracting the anodic branch of Cycle 2 from Cycle 1. The shaded areas indicate the integrated CO oxidation charge, and the corresponding electrochemically active Pt areas are reported in the legend.

8 LEIS spectra of the silica-coated edep-Pt|Ti surface before and after long CO exposure

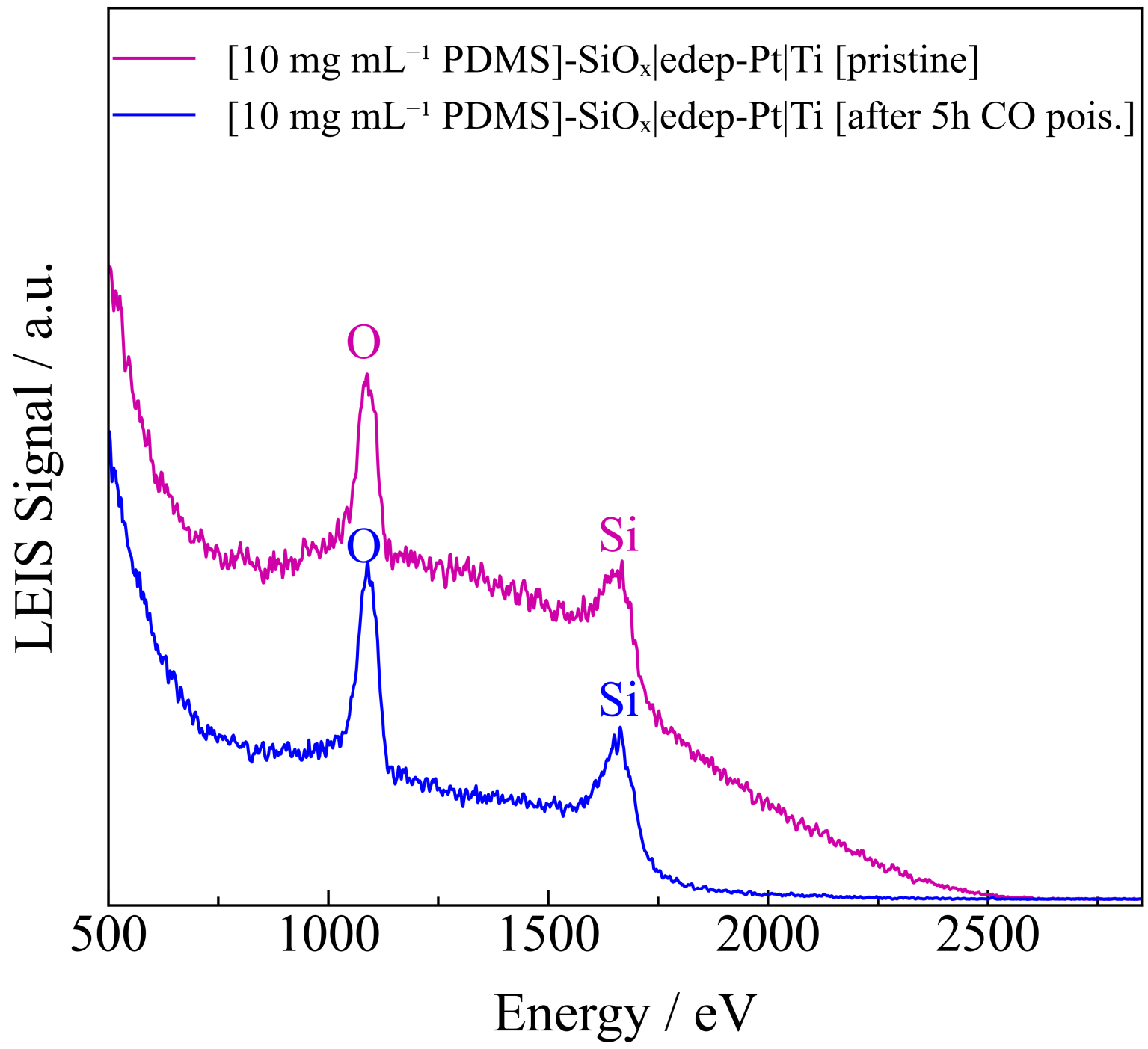

Figure S14. Low-energy ion scattering (LEIS) spectra for [10 mg mL^{-1} PDMS]-SiO_x|edep-Pt|Ti measured in the pristine state and after 5 h of CO-poisoning exposure. The only peaks present are assigned to O and Si and after the long CO poisoning experiment the tested silica overlayer still encapsulates the Pt nanoparticles and keeps its conformality as the pristine one (only the background shifts but no exposed Pt is visible).

9 Non-linear CO poisoning model fitting and analysis

The CO poisoning experiment that is conducted involves the start of a constant CO flow at t_0, during HOR driven at 0.15V (RHE) in the H_2 saturated acidic electrolyte, at 2500 rpm. Therefore, the CO concentration is evolving simultaneously with any CO poisoning taking place once any amount of CO reached the Pt-electrolyte interface. When a Pt working electrode is placed into a continuously CO-purged, acidic aqueous electrolyte, the dissolved CO concentration at the interface rises toward a finite solubility limit. Assuming well mixed conditions (rotation experiment) and gasliquid mass transfer being not rate-limited by interfacial area changes, the bulk dissolved CO concentration follows a first-order approach to saturation after the switch at t_0=0:

$$C(t) = C_\infty \left(1 - e^{-t/\tau_m}\right) \tag{S10}$$

where τ_m is the mixing/gas-exchange time constant and C_∞ is the equilibrium (asymptotic) dissolved CO concentration the liquid approaches after the switch on of CO bubbling. For the ideal, well-mixed system we assume, it is basically defined by Henrys law, i.e. by the CO partial pressure. Simplifying our model, we can define the normalized bulk dissolved CO concentration rise and make our model non-dependent on the C_∞ value, i.e. the applied CO flowrate and partial pressure:

$$c(t) = 1 - e^{-t/\tau_m} \tag{S11}$$

The Pt electrode poisoning (loss of Pt active sites) is driven not only by the instantaneous CO concentration but by the cumulative CO exposure. Again for a well-mixed system, the normalized CO exposure variable E can be defined as:

$$E(t) = \int_0^t c(t')\,\mathrm{d}t' = t + \tau_m\left(e^{-t/\tau_m} - 1\right) \tag{S12}$$

CO poisoning of Pt often proceeds by affecting two types of Pt active site populations, one is the high-energy sites (defects, step edges, kinks) that block rapidly, and the other could be terrace or secondary sites that block more slowly. This CO poisoning mechanism of the two-population Pt sites can be represented via two JohnsonMehlAvramiKolmogorov (JMAK) processes acting in a parallel fashion.[4,5] By defining population 1 and population 2 as the fast and slow CO blocking sites respectively, we have the JMAK term-process 1 and 2 respectively. Assuming that these two distinct populations poison independently, we need to introduce a p-weighted sum of the two JMAK terms, since the contribution of each population being poisoned by CO is unknown. The CO poisoning curves are the result of potentiostatic experiments (chronoamperometry), thus the response variable is current i versus time t. Having this we can define the full proposed model for CO poisoning:

$$i(t) = i_\infty + \left(i_0 - i_\infty\right)\left\{p\,\exp\left[-\left(\frac{E(t)}{\tau_1}\right)^{n_1}\right] + (1-p)\,\exp\left[-\left(\frac{E(t)}{\tau_2}\right)^{n_2}\right]\right\} \tag{S13}$$

- $i(t)$ measured current response (mA), t in seconds.
- i_0, i_∞ initial and long-time current asymptotes: fully active and fully poisoned limits in the chosen experimental protocol-parameters.
- $p \in [0,1]$ weight fraction of the total contribution of population 1, with the balance $1-p$ weighing population 2.

- $\tau_1,\ \tau_2 > 0$ exposure time scales or time constants of the two JMAK processes (with $E(t)$ normalized, units are seconds).

- $n_1,\ n_2 > 0$ Avrami exponents, reflecting CO nucleation/growth and blocking geometry of the Pt sites.

- $E(t)$ normalized cummulative CO exposure (Equation S12).

We next focus exclusively on how the silica overlayer on the 10 mg mL^{-1} SiOx|edep-Pt|Ti electrode modifies the poisoning dynamics of Pt under CO. We quantify the time-dependent deactivation of Pt sites under potentiostatic HOR (chronoamperometry) at 0.15 V vs RHE (2500 rpm) in 1,700 ppm CO/H_2, using the two-population Johnson–Mehl–Avrami–Kolmogorov (JMAK) CO-poisoning kinetics model derived, to separate intrinsic surface blocking from the finite time required to establish steady dissolved CO. Figure S15 compares the experimental potentiostatic CO-poisoning transients of uncoated edep-Pt|Ti (yellow symbols) and the 10 mg mL^{-1} SiO_x|edep-Pt|Ti electrode (red symbols). Upon CO introduction at 1,700 ppm, the uncoated edep-Pt|Ti curve shows a short induction followed by a very steep decay, dropping from its initial value to near-zero current within ∼2000–3000 s, indicating rapid accumulation of adsorbed CO that blocks most of the HOR-active Pt sites. In contrast, the coated electrode deactivates much more gradually. The current density decreases smoothly over hours, from ∼2.5 to ∼1.5 mA cm^{-2} in ∼5 h and does not approach full shutdown. The slow, nonzero long-time response indicates that a fraction of Pt remains effectively protected by the silica overlayer, enabling sustained HOR under this continuous CO exposure.

Polycrystalline Pt surfaces commonly exhibit at least two kinetically distinct site classes for CO adsorption (e.g., terraces vs defect-rich regions), producing an initially rapid loss followed by a slower decay to a long-time limit. In the model, the measured current density $j(t)$ is expressed as a p-weighted sum of two independent JMAK decays (populations 1 and 2), driven by a normalized cumulative CO exposure $E(t)$ which models the finite time required to establish a steady dissolved CO. The Avrami exponents n_1 and n_2 control whether the Pt site activity decay is closer to simple site blocking ($n \sim 1$) or more cooperative/nucleation-growth-like ($n > 1$), while τ_1 and τ_2 set the characteristic poisoning timescales.[4,5] Notably, a crucial experimental constraint included in the analysis is the finite time required for dissolved CO in the electrolyte to reach steady state after switching on the CO/H_2 feed. We fix the CO build-up timescale $\tau_m \approx 300$ s (∼5 min), which prevents over-interpreting the earliest-time current drop as purely surface kinetics when part of the transient is mass-transfer controlled.

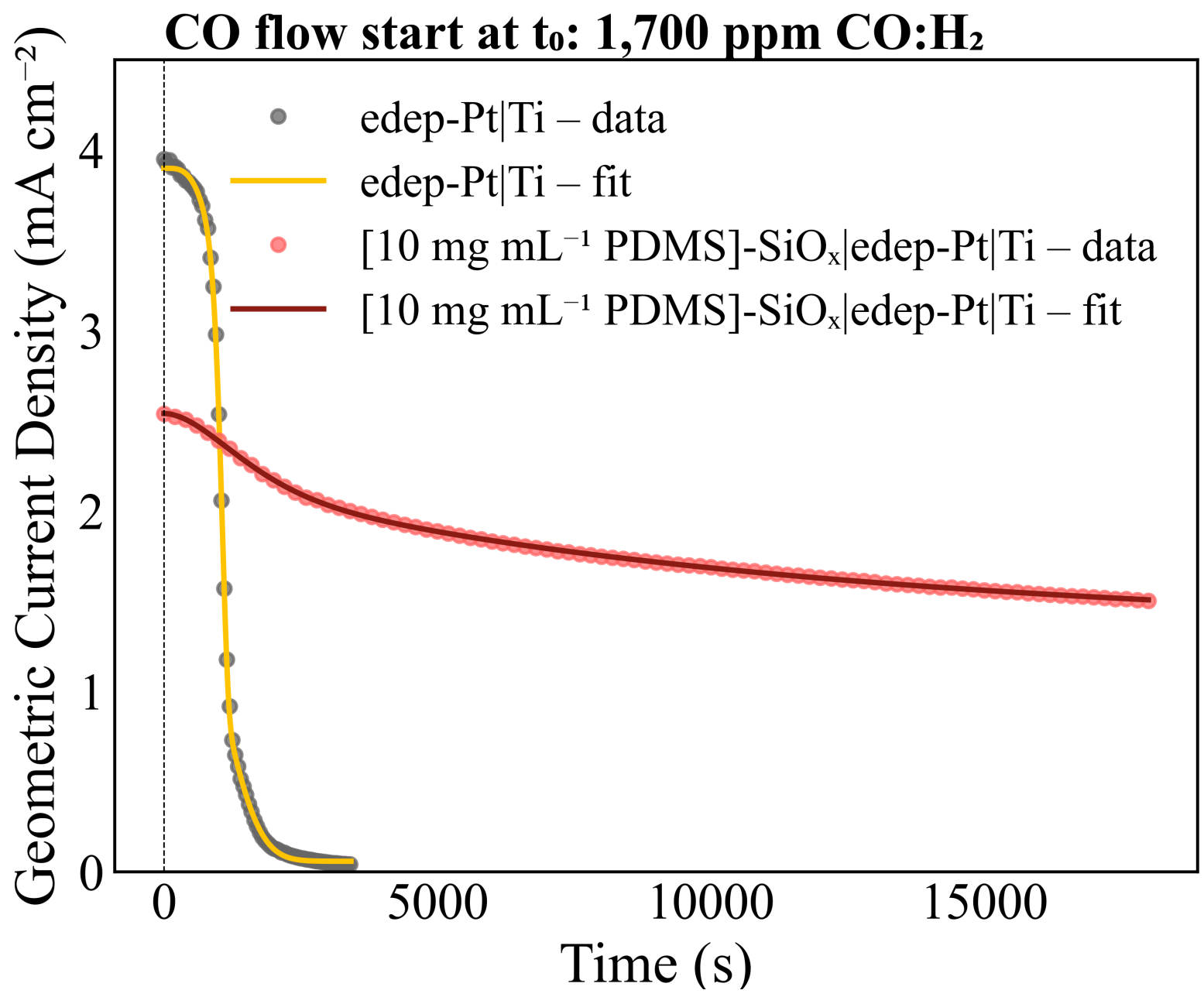

Figure S15. Potentiostatic CO poisoning kinetics on uncoated and optimally coated structured Pt with JMAK-model fits: Experimental chronoamperometry traces (symbols) and fits (lines) using the two-population, exposure-based JMAK model for uncoated edep-Pt|Ti and the optimal 10 mg mL^{-1} SiO_x|edep-Pt|Ti electrode under CO poisoning (CO introduced at t_0 at 1,700 ppm, 0.15 V vs. RHE and 2500 rpm). Electrolyte: 0.1 M $HClO_4$ (pH = 1.1).

Fitting the model to the poisoning curves in Figure S15 with near-zero residuals (Figure S16), the results in Table S1 highlight two key effects of the SiO_x overlayer on structured Pt. First, the coated electrode does not approach a near-zero current asymptote as the uncoated one and instead, it retains a substantial long-time current ($i_\infty \approx 1.31$ vs. ≈ 0.06 mA), consistent with persistent HOR under constant CO exposure. Second, the slow poisoning regime is dramatically decelerated by the presence of the SiOx coating (τ_2 increases from $\sim 10^3$ s to $\sim 1.1 \times 10^4$ s), indicating that it suppresses the continued CO poisoning of less accessible Pt sites or the progressive CO domain growth. In parallel, the fitted Avrami exponents decrease for the coated electrode, with both n_1 and n_2 approaching unity. Within this phenomenological framework, this corresponds to an apparent decay behavior closer to first-order site blocking, whereas the uncoated electrode exhibits a faster-than-exponential, cooperative-like transient[5]. These fitted exponents describe the macroscopic current-decay behavior and should not be interpreted as direct evidence for a change in the intrinsic adsorption mechanism.

Table S1. Fitted parameters for the two-population JMAK (p-weighted) model applied to the experimental CO poisoning curves of Figure S15.

Parameter	edep-Pt|Ti	[10 mg mL^{-1}]-SiO$_x$|edep-Pt|Ti
i_0 (mA)	3.90	2.54
i_∞ (mA)	0.06	1.31
p (-)	0.61	0.25
τ_1 (s)	788	1595
n_1 (-)	8.3	1.6
τ_2 (s)	1032	10956
n_2 (-)	2.2	0.9
τ_m (s) [fixed]	300	300

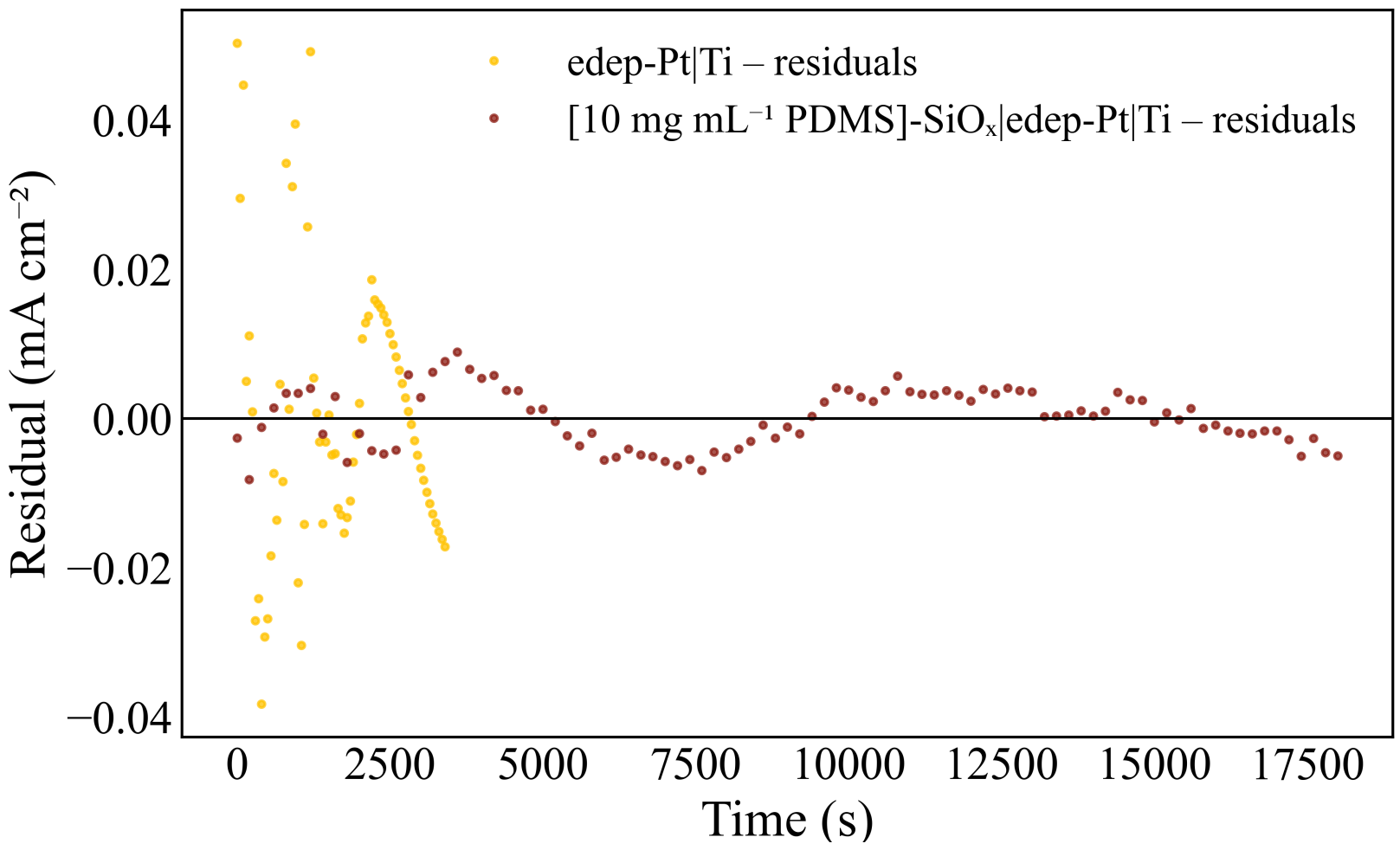

Figure S16. Residuals (defined as $i_{exp} - i_{fit}$, plotted as current density) are shown as a function of time for CO-poisoning chronoamperometry datasets measured under HOR conditions (potentiostatic hold at 0.15 V vs. RHE in H_2-saturated acidic electrolyte, 2500 rpm), where a constant CO flow is switched on at $t_0 = 0$ and the dissolved CO concentration rises toward saturation. Datasets correspond to edep-Pt|Ti and [10 mg mL^{-1} PDMS]-SiO$_x$|edep-Pt|Ti electrodes. The near-zero, structure-minimized residuals quantify the goodness-of-fit over the full poisoning duration.

10 CO-poisoning CA of atomic layer deposition (ALD) SiO_2 on edep-Pt|Ti

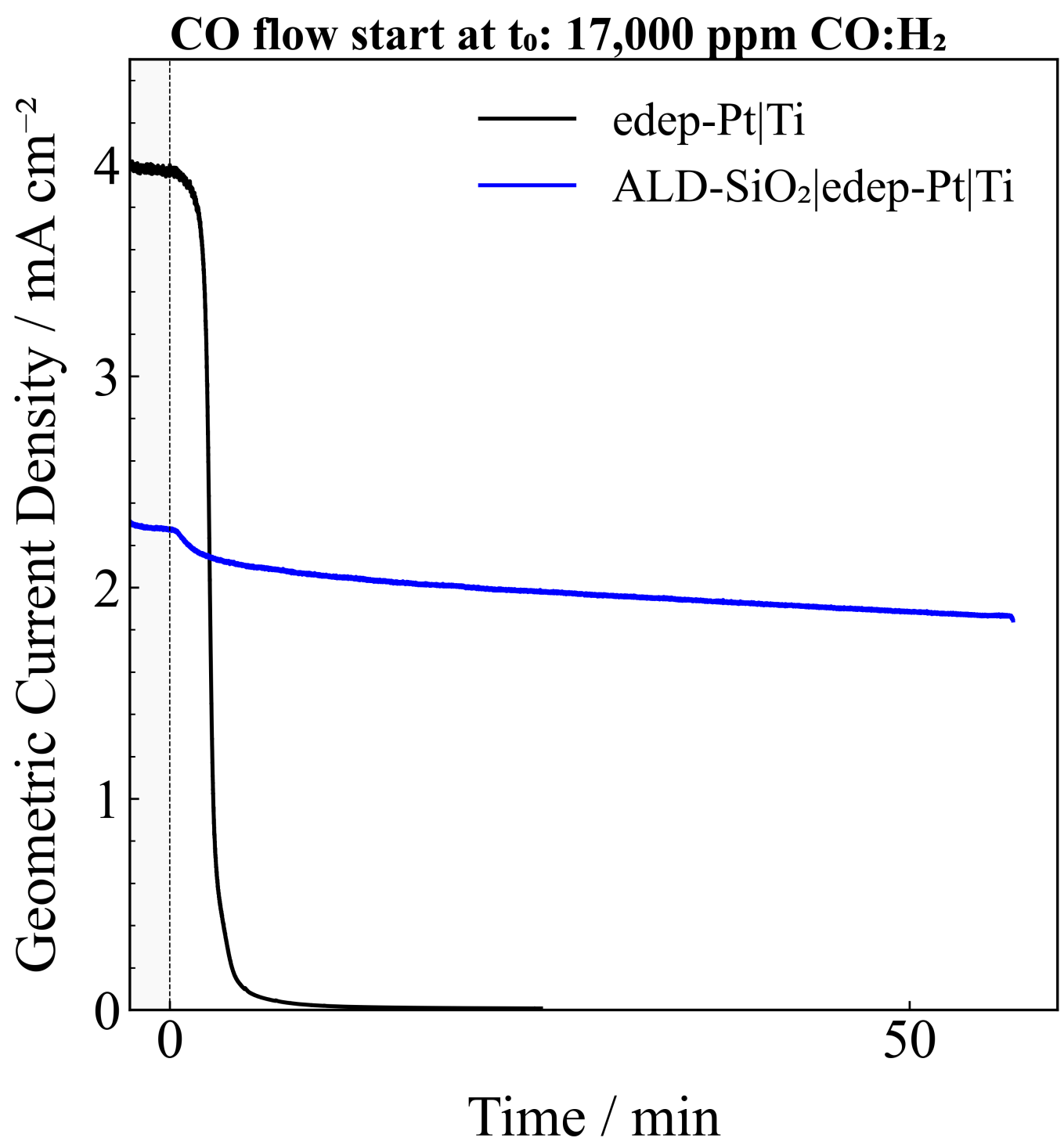

Figure S17. CO-poisoning HOR chronoamperometry of a ALD-SiO_2|edep-Pt|Ti sample at 0.15 V vs. RHE (2500 rpm): electrode is operated in pure H_2 for 3 min, then CO is introduced at t_0 to obtain 17,000 ppm CO in H_2. Electrolyte for all measurements: 0.1 M $HClO_4$ (pH = 1.1) at ambient conditions.

References

[1] H. Frei and D. V. Esposito, eds. *Ultrathin Oxide Layers for Solar and Electrocatalytic Systems*. Royal Society of Chemistry, 2022. ISBN: 9781839161797.

[2] M. E. S. Beatty, E. I. Gillette, A. T. Haley, and D. V. Esposito. “Controlling the Relative Fluxes of Protons and Oxygen to Electrocatalytic Buried Interfaces with Tunable Silicon Oxide Overlayers”. In: *ACS Applied Energy Materials* 3.12 (Dec. 28, 2020), pp. 12338–12350. ISSN: 2574-0962, 2574-0962. DOI: 10.1021/acsaem.0c02359.

[3] J. A. Bau and K. Takanabe. “Ultrathin Microporous SiO_2 Membranes Photodeposited on Hydrogen Evolving Catalysts Enabling Overall Water Splitting”. In: *ACS Catalysis* 7.11 (Nov. 3, 2017), pp. 7931–7940. ISSN: 2155-5435, 2155-5435. DOI: 10.1021/acscatal.7b03017.

[4] M. Fanfoni and M. Tomellini. “The Johnson-Mehl- Avrami-Kohnogorov model: A brief review”. In: *Il Nuovo Cimento D* 20.7 (July 1998), pp. 1171–1182. ISSN: 0392-6737, 1826-9893. DOI: 10.1007/BF03185527.

[5] O. Pedroso, Y. Champion, W. Botta, and G. Zepon. *Johnson-Mehl-Avrami-Kolmogorov model applied to describe the site blocking effect in interstitial solid solution*. ChemRxiv preprint. Oct. 2023. DOI: 10.26434/chemrxiv-2023-8d22n.
